# Discovery and Optimization of AMT‐676, a CDH17‐Targeting ADC for the Treatment of Advanced Gastrointestinal Cancers

**DOI:** 10.1002/advs.77521

**Published:** 2026-09-09

**Authors:** Ying‐nan Wang, Dan‐yun Ruan, Jing Shi, Yue Huang, Hui Sheng, Ya‐ling Huang, Linjie Ma, Lijun Wang, Caiwei Chen, Yixuan Wang, Yue Zhang, Yi Shen, Qing Zhang, Jianjian Zhang, Yanfang Tang, Jin‐ling Zhang, Jia‐jia Hu, Chen‐yi Wu, Jia‐ying Chen, Xun Meng, Shu‐Hui Liu, Feng Wang

**Affiliations:** ^1^ Department of Medical Oncology State Key Laboratory of Oncology in South China Guangdong Provincial Clinical Research Center For Cancer Sun Yat‐Sen University Cancer Center Sun Yat‐Sen University Guangzhou People's Republic of China; ^2^ Research Unit of Precision Diagnosis and Treatment for Gastrointestinal Cancer Chinese Academy of Medical Sciences Guangzhou People's Republic of China; ^3^ Department of Clinical Research State Key Laboratory of Oncology in South China Guangdong Provincial Clinical Research Center For Cancer Sun Yat‐sen University Cancer Center Sun Yat‐sen University Guangzhou People's Republic of China; ^4^ Multitude Therapeutics Shanghai People's Republic of China; ^5^ State Key Laboratory of Oncology in South China Sun Yat‐sen University Cancer Center Guangzhou People's Republic of China; ^6^ Abmart Shanghai People's Republic of China; ^7^ Multitude Therapeutics Redwood City California USA

**Keywords:** antibody‐drug conjugates, cadherin 17, gastrointestinal cancers

## Abstract

Advanced‐stage gastrointestinal (GI) cancers present an unmet need for innovative therapies, and antibody‐drug conjugates (ADCs) offer promising solutions. This study investigates the antitumor efficacy and toxicity of AMT‐676, a novel ADC targeting Cadherin 17 (CDH17), in GI cancers. Utilizing MabArray screening and immunohistochemistry, CDH17 was identified as a promising ADC target in GI tumors with minimal expression in healthy organs. The ADC was optimized through comprehensive in vitro and in vivo evaluations of binding affinity, cytotoxicity, pharmacokinetics, and toxicity, with antitumor potential evaluated using cell line‐derived (CDX) and patient‐derived tumor xenograft (PDX) models. Leveraging the T moiety‐exatecan platform, we synthesized AMT‐676, comprising a high‐affinity CDH17‐specific antibody, a hydrophilic self‐immolative T1000 linker, and exatecan with a Drug‐to‐Antibody Ratio (DAR) of 4. AMT‐676 demonstrated sustained antitumor responses across CDX and PDX GI models with diverse CDH17 expression, notably inhibiting metastatic growth in a colorectal cancer model. Cynomolgus monkey studies revealed favorable pharmacokinetics and manageable toxicity associated with AMT‐676. In conclusion, we developed a novel CDH17‐targeting ADC characterized by a high therapeutic index and tolerable toxicity profile, highlighting its promise for GI cancer treatment. A first‐in‐human, phase I clinical trial of AMT‐676 in patients with advanced solid tumors is currently underway (NCT06400485).

## Graphical abstract

AMT‐676 is a novel antibody‐drug conjugate targeting CDH17, an adhesion molecule uniquely exposed on gastrointestinal tumor surfaces. Engineered with an optimized exatecan payload, it demonstrates profound tumor regression and a robust bystander effect across diverse preclinical models. Exhibiting a highly favorable therapeutic index, AMT‐676 emerges as a promising targeted breakthrough for advanced gastrointestinal malignancies.

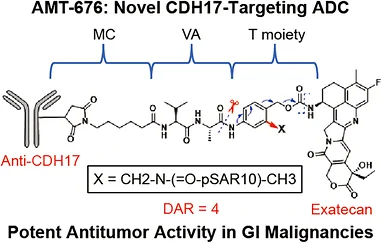

## 1 Introduction

Gastrointestinal (GI) cancers encompass a heterogeneous group of diseases affecting the digestive system, including esophageal cancer, gastric cancer (GC), hepatocellular carcinoma, pancreatic cancer, biliary tract cancer (BTC), colorectal cancer (CRC), anal cancer, and neuroendocrine tumors (NETs) [1]. These cancers are prevalent worldwide and account for a significant proportion of cancer‐related deaths globally. In the United States, GI cancers represented 17.9% of new cancer cases and accounted for 28.3% of cancer‐related deaths in 2022 [2]. Global analyses suggest that approximately one in 12 individuals will develop GI cancers, with one in 16 succumbing to these diseases [3]. CRC, in particular, stands out as the most commonly diagnosed GI cancer, with an annual incidence exceeding 1.80 million cases globally according to the World Health Organization (WHO) [4]. In China, CRC has emerged as the predominant GI cancer, with 517 100 new cases reported in 2022 [5]. For CRC and other GI cancers in the curative setting, the mainstay therapeutic modalities include surgical resection, neoadjuvant or adjuvant chemotherapy, and radiotherapy, often used in combination. For advanced disease, targeted therapies and immunotherapy have been integrated into standard regimens alongside systemic chemotherapy. However, these treatments face significant challenges and limitations, underscoring the critical need for novel and effective anticancer strategies.

Over the past decade, antibody‐drug conjugates (ADCs) have emerged as a novel class of anticancer agents that are effective against a broad spectrum of solid tumors and hematological malignancies [6, 7]. ADCs consist of antibodies, linkers, and cytotoxic payloads specifically designed to selectively target tumor cells. This targeted approach enhances therapeutic efficacy and reduces toxicity by minimizing systemic drug distribution [8, 9]. Upon binding to tumor‐specific or tumor‐associated antigens, ADCs are internalized into tumor cells where the linker is cleaved by low pH environment or tumor‐specific enzymes, releasing the cytotoxic drug and inducing cell death [10, 11]. Additionally, ADCs potentially exert a “bystander effect” by releasing cytotoxic payloads into the tumor microenvironment, which can eliminate neighboring tumor cells, even those with low antigen expression levels [12]. As of early 2024, the FDA has approved 15 ADCs for oncology therapeutics [7]. However, only a limited number of these ADCs have been utilized in the treatment of GI cancers, underscoring significant unmet clinical needs in this area.

Cadherin 17 (CDH17) is a member of the cadherin superfamily and serves as a principal adhesion molecule at intercellular junctions, which are crucial for maintaining tissue structure and morphology [13]. Unlike classic cadherins like E‐, N‐, and P‐cadherins, CDH17 features seven extracellular cadherin repeats rather than the typical five, a single transmembrane domain, and a short cytoplasmic domain of only 20 amino‐acid residues, in contrast to the usual 150–160 residues [14]. Initially identified in rat polarized epithelia of the liver and intestine, it was designated as liver‐intestine cadherin (LI‐cadherin) [15]. However, in humans and mice, CDH17 expression is predominantly restricted to epithelial cells of the small intestine, colorectum, and pancreatic ducts, with expression rarely detected in the liver and stomach [16, 17]. Dysregulation of CDH17 expression levels and patterns has been observed in various human cancers. Notably, CDH17 is widely expressed in digestive system tumors such as colorectal, gastric, esophageal, and pancreatic cancers, as well as cholangiocarcinoma. It is also expressed in female reproductive system tumors such as ovarian and cervical cancers, but is nearly absent in other systemic tumors [17, 18, 19]. Remarkably, almost all the cases of CRC express CDH17, underscoring its potential role in CRC therapeutics development. Furthermore, CDH17 is expressed in certain NETs, including small‐cell lung cancer and specific digestive system NETs [20, 21, 22]. CDH17 exhibits distinct expression patterns between cancerous and normal tissues, similar to another adhesion molecule targeted by ADCs, claudin18.2 [23]. In normal cells, CDH17 is localized exclusively to the lateral membrane where tight junctions form and is absent from apical or basal membranes, making it inaccessible in healthy tissues. Conversely, CDH17 becomes exposed on the surface of tumor tissues due to loss of cellular polarity [24]. This unique feature positions CDH17 as a promising candidate for targeted ADC therapies.

In this study, we designed AMT‐676, a T moiety‐exatecan‐based [25] ADC targeting CDH17, which demonstrated potent antitumor efficacy against CDH17‐expressing solid tumors. AMT‐676 exhibited strong antitumor activity across cell lines, CDX, and PDX models of GI cancers. Pharmacokinetic and toxicological studies conducted in monkeys revealed favorable terminal half‐life and stability of AMT‐676, and more importantly, high tolerability and compelling safety profiles in treated animals. Our findings suggest that targeting CDH17 with ADCs may represent a promising new therapeutic approach for treating GI tumors.

## 2 Results

### 2.1 Identification of CDH17 as an ADC Target for CRC

To identify potential antibody targets for CRC, we employed MabArray screening to detect membrane proteins in the SW480 CRC cell line (Figure S1A) using the procedure detailed previously [26]. Among the antibodies screened, CL‐676 exhibited the strongest binding affinity in flow cytometry, and was selected for further investigation. Immunoprecipitation‐mass spectrometry (IP‐MS) analysis identified CDH17 as the potential binding partner of CL‐676 in SW480 cells (Figure S1B,C). Co‐localization studies of CL‐676 with GFP‐tagged CDH17 in 293T cells confirmed this interaction (Figure S1D). CDH17 acts as an adhesion protein and has been recognized as a novel diagnostic marker for GI cancers [18]. Notably, while CDH17 is surface‐exposed on tumor tissues, it remains concealed beneath tight junctions in normal tissues, allowing for selective ADC targeting of tumor cells and minimizing toxicity to healthy tissues [18, 27]. Currently, there are no marketed ADCs targeting CDH17, underscoring its untapped therapeutic potential.

**FIGURE 1:**
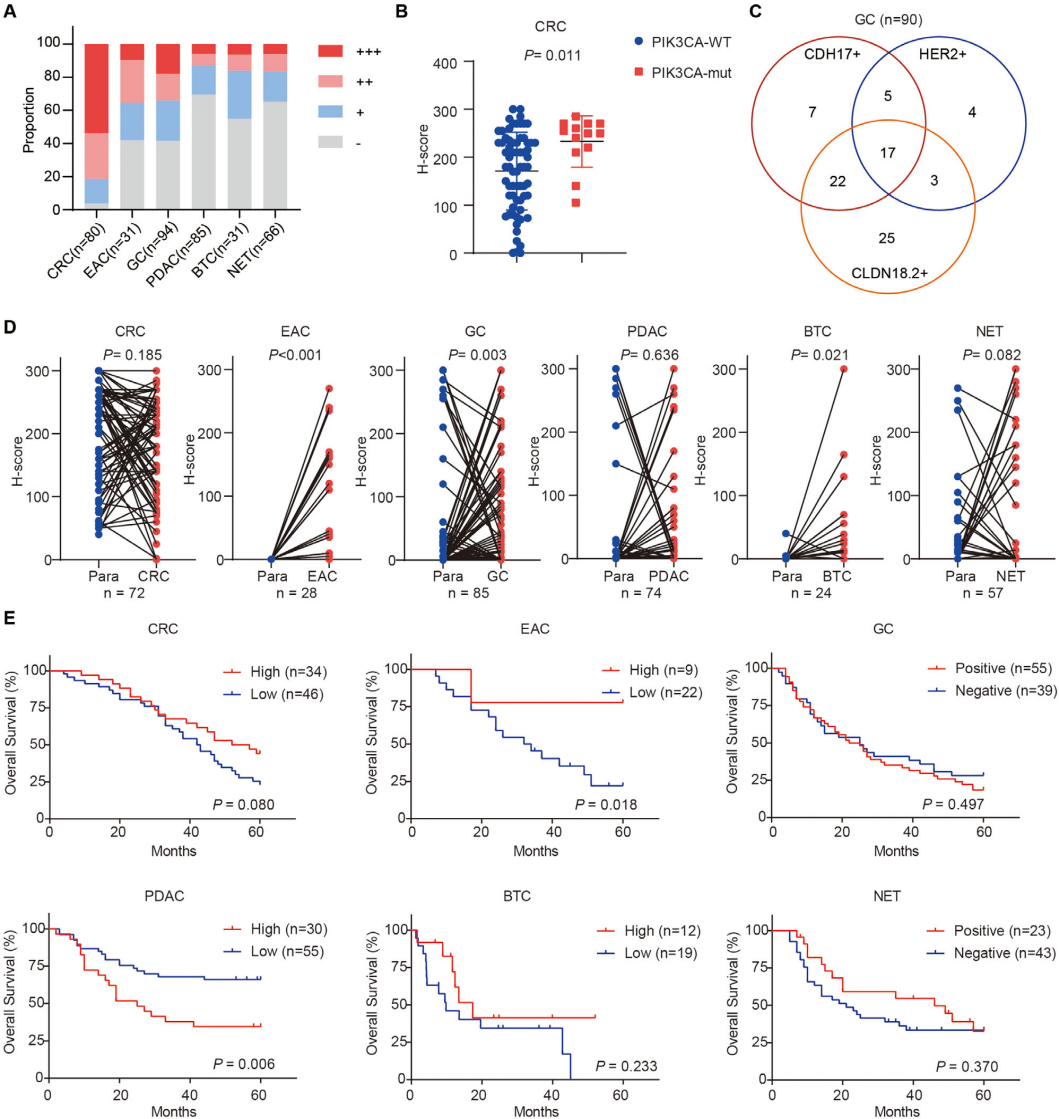
Nomination of CDH17/clone 814 as a target/antibody pair for constructing ADCs. (A) Tumor tissue samples from GI cancers, including CRC, EAC, GC, PDAC, BTC and NET, were collected from Sun Yat‐sen University Cancer Center. CDH17 expression was evaluated using IHC staining with the number (*n*) of patient samples examined indicated. CDH17 positivity was defined by the threshold of H‐score > 10. (B) In the CRC cohort in (A), we compared CDH17 expression between the PIK3CA wild‐type (WT) and PIK3CA mutant subgroups. (C) The Venn diagram illustrates the relationship among CDH17, HER2 and CLDN18.2 expression within the GC cohort from (A). IHC staining scores of 1+ or higher are considered indicative of positive expression. (D) A comparison of CDH17 expression levels between tumor regions and peritumoral regions is displayed. (E) Kaplan‐Meier analysis shows OS in GI cancers associated with high and low CDH17 expression levels. The cutoff value for grouping CDH17 expression was determined using the area under the curve (AUC) from receiver operating characteristic (ROC) analysis. Data are represented as mean ± SD. Statistical analyses were performed using Student's t‐test (B and D) and log‐rank test (E).

To evaluate the suitability of CDH17 as a target for ADC development, we conducted immunohistochemical (IHC) staining across various healthy human organs. To find the most robust antibody for detecting CDH17 in IHC assays, we first screened multiple mouse anti‐Human CDH17 monoclonal antibodies and found that clone 814 gave strong membranous staining in epithelial cells in human normal colon and colorectal cancer samples, which are known to have CDH17 expression [19]. Therefore, clone 814 was subsequently developed for IHC assays. The staining in healthy human organs is predominantly localized to the GI tract, particularly in the small intestine and colon, with sporadic weak expression in the stomach (Figure S2A,B). We then performed IHC studies on different types of GI cancer surgically resected samples from Sun Yat‐sen University Cancer Center, including CRC, esophageal adenocarcinoma (EAC), GC, pancreatic ductal adenocarcinoma (PDAC), BTC and NETs (Figure 1A and Figure S3). In our analysis of CRC patients, 77 out of 80 (96.3%) tumor tissue samples showed positive CDH17 expression, aligning with previous findings [17, 18]. The positive rates for CDH17 expression in other GI cancers were found to be 58.1% in EAC, 58.5% in GC, 30.6% in PDAC, 45.2% in BTC, and 34.8% in NETs. Further, we explored the correlation between CDH17 expression and genetic profiles in CRC by analyzing high‐throughput sequencing data from the CRC cohort presented in Figure 1A. Among over 1000 genes analyzed, CDH17 levels were significantly elevated in patients with PIK3CA mutations, indicating potential therapeutic relevance in this subgroup (Figure 1B). In the context of GC, we investigated the association among the expressions of CDH17, HER2 and CLDN18.2. Our results revealed that among HER2‐positive specimens, a high proportion (75.9%, 22 out of 29 cases) showed CDH17 positivity. In CLDN18.2‐positive specimens, 58.2% (39 out of 67 cases) were positive for CDH17 (Figure 1C). Notably, a considerable number of HER2‐negative (47.5%, 29 out of 61 cases) or CLDN18.2‐negative (52.2%, 12 out of 23 cases) GC samples still exhibited CDH17 positivity. This highlights the potential of CDH17 targeting in HER2/CLDN18.2 therapy‐ineligible patients. Additionally, we evaluated CDH17 expression levels in peritumoral regions. The results indicated that CDH17 expression was significantly higher in tumor tissues in EAC, GC, and BTC compared to adjacent normal tissues, while such difference did not reach statistical significance in CRC, PDAC, and NETs (Figure 1D). Despite the recognition of CDH17's role, its relationship with prognosis in cancer remains unclear. To address this, we conducted survival analyses using optimal CDH17 level cut‐offs to assess its association with overall survival (OS) across different GI cancers. Elevated CDH17 levels were associated with poor prognosis in PDAC and good prognosis in EAC. High CDH17 levels seemed to show a favorable trend in prognosis for CRC, BTC, and NETs though not statistically significant. No clear correlation was found between CDH17 levels and OS in GC (Figure 1E).

### 2.2 Development and Evaluation of AMT‐676: A Novel CDH17‐Targeting ADC

**FIGURE 2:**
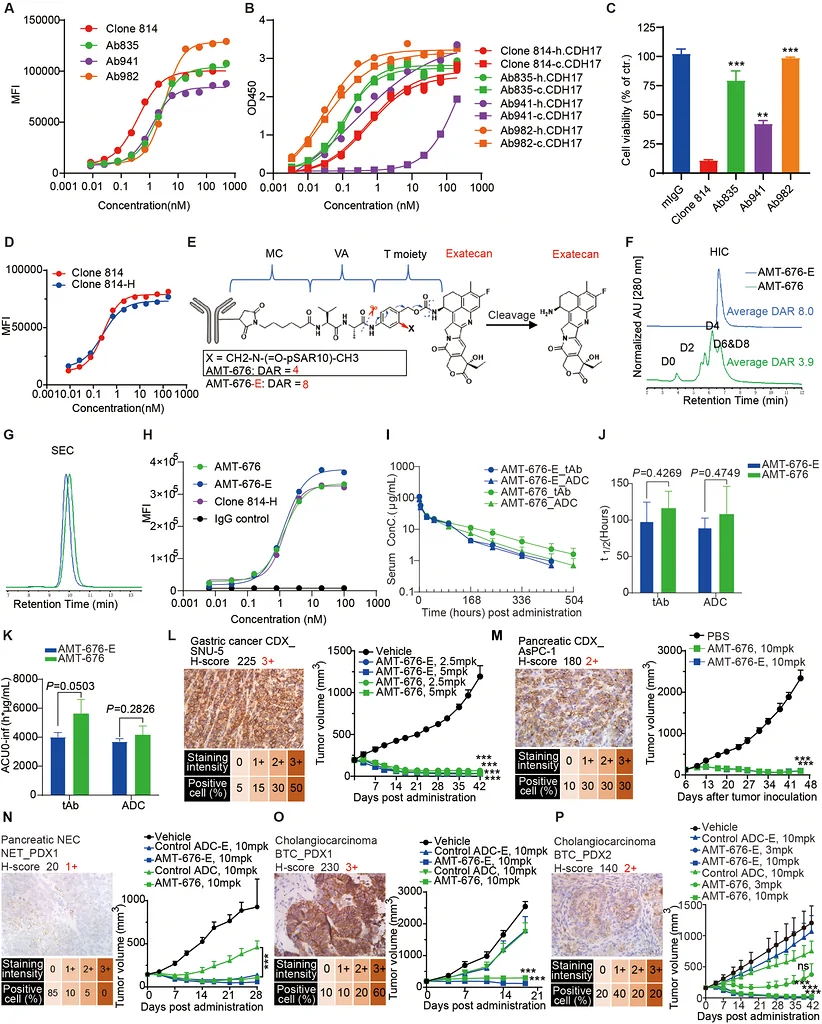
Design and efficacy of CDH17‐targeting ADCs. (A) The cell binding affinity of four CDH17‐targeting antibodies was assessed using flow cytometry. (B) Binding affinity of CDH17‐targeting monoclonal antibodies (mAbs) on recombinant CDH17 proteins from human and cynomolgus monkey quantified via ELISA. (C) 1 nM of CDH17 targeting mAbs was mixed with 5 nM vc‐MMAE‐conjugated anti‐mouse IgG secondary antibody and incubated with COLO205 cells in 96‐well plates for three days. Endocytosis‐mediated cytotoxicity was subsequently measured using the CCK8 assay. (D) Flow cytometry revealed the cell binding affinity of the humanized anti‐CDH17 mAb (clone 814‐H) compared to its parental mAb (clone 814) on CDH17‐positive COLO205 cells. (E) Structural representation of AMT‐676 and AMT‐676‐E. Exatecan is conjugated to T1000, a polysarcosine sidechain (indicated in red), which is modified with a self‐immolative PABC spacer (denoted by a red scissor). T1000‐exatecan is subsequently linked to clone 814‐H via the VA‐MC linker, facilitating the release of exatecan as the cytotoxic payload. AMT‐676 exhibits an average drug‐to‐antibody ratio (DAR) of 4, while AMT‐676‐E shows an average DAR of 8. (F) Hydrophobic Interaction Chromatography (HIC) profiles of AMT‐676 and AMT‐676‐E are presented. The average DAR, determined through reverse‐phase chromatographic analysis, is indicated. (G) Size Exclusion Chromatography (SEC) profiles of AMT‐676 and AMT‐676‐E. Purity levels for AMT‐676 and AMT‐676‐E are quantified at 99.08% and 99.13%, respectively. (H) Flow cytometry demonstrates the impact of conjugating T1000‐exatecan to clone 814‐H on its binding affinity for the CDH17‐positive tumor cell line COLO205. (I) Pharmacokinetic evaluation of AMT‐676 and AMT‐676‐E conducted in Balb/c nude mice. Mice (*n* = 3) were administered 5 mg kg^−1^ of either AMT‐676 or AMT‐676‐E via intravenous injection on day 0, with subsequent measurement of total antibody and ADC concentrations in plasma at specified time points. (J) Half‐life assessment of AMT‐676 and AMT‐676‐E in mice. (K) The AUC values for AMT‐676 and AMT‐676‐E following administration in mice. (L‐P) In vivo efficacy of AMT‐676 and AMT‐676‐E evaluated in GC CDX (L), PDAC CDX (M), Pancreatic NEC PDX (N) and Cholangiocarcinoma PDX (O,P) models (*n* = 5). The same dose of AMT‐676, AMT‐676‐E was administered intravenously when tumor size reached an average of 150–200 mm^3^. CDH17 expression in the CDXs was shown via IHC imaging of untreated mouse tumor tissue, accompanied by H‐scores. Statistical comparisons: For 2L–M: each treatment group vs. Vehicle or PBS group; For 2N–P: AMT‐676 vs. Control ADC (Ritu‐T1000‐Exatecan(DAR4)), and AMT‐676‐E vs. Control ADC‐E (Ritu‐T1000‐Exatecan(DAR8)). Data are represented as mean ± SD (C, I, J and K) or mean ± SEM (L‐P). Statistical analyses were performed using one‐way ANOVA (C), Student's t‐test (J and K) and two‐way ANOVA followed by Tukey's multiple‐comparisons test (L‐P). **, *p* < 0.01; ***, *p* < 0.001; ns, not significant difference.

To design the most effective ADC targeting CDH17, we further generated multiple anti‐CDH17 antibodies and assessed their binding affinity in CRC cells using flow cytometry. Among the four antibodies tested, clone 814, the same antibody that works best in our IHC assay, demonstrated the highest affinity (EC_50_ = 0.40 nM) for COLO205 cells (Figure 2A). We proceeded to investigate the recognition of cynomolgus monkey CDH17 (c.CDH17) by these antibodies and found clone 814 exhibited similar binding affinity for c.CDH17 as it did for human CDH17 (h.CDH17) (Figure 2B). Subsequently, we evaluated the cytotoxic potential of the antibodies by utilizing a vc‐MMAE conjugated anti‐mouse IgG antibody as a secondary conjugate for cell‐killing assays [28]. Among the antibodies tested, clone 814 displayed the strongest cytotoxicity against cancer cells compared to the other candidates (Figure 2C) and was then humanized for future clinical application by grafting the CDRs onto the closed human germline [29]. The binding affinity of the humanized antibody, designated as clone 814‐H, was confirmed using flow cytometry on COLO205 cells (Figure 2D). Clone 814‐H was ultimately selected as the lead clinical candidate for ADC design targeting CDH17.

Following the selection of the CDH17‐targeting antibody clone 814‐H, we then focused on optimizing the linker and cytotoxic payload design. Leveraging the previously established linker‐payload platform, T1000‐exatecan [25], we successfully conjugated this linker‐payload to clone 814‐H to create a novel ADC, named AMT‐676. With the aim of optimizing the ADC design to expand the therapeutic window, we evaluated the potency and tolerability of two ADCs with different drug‐to‐antibody ratios (DAR): AMT‐676 with a DAR of 4 and AMT‐676‐E with a DAR of 8 (Figure 2E,F). High purity was obtained with both ADC formats, as confirmed by size exclusion chromatography (SEC) analysis (Figure 2G). Flow cytometry results indicated that the conjugation of T1000‐exatecan to clone 814‐H did not compromise its binding affinity to CDH17 in COLO205 cells (Figure 2H).

We subsequently assessed the in vivo pharmacokinetics of AMT‐676 and AMT‐676‐E in Balb/c nude mice. The plasma concentration profiles are largely similar between AMT‐676 and AMT‐676‐E for both total antibody (tAb) and ADCs (Figure 2I), with higher concentration and exposure generally observed for AMT‐676. The half‐lives (T_1/2_) of AMT‐676 and AMT‐676‐E are 108.1 ± 38.3 h and 89.7 ± 13.0 h, respectively (Figure 2J,K; Tables S1 and S2).

To further evaluate the therapeutic efficacy of these ADCs, we conducted in vivo experiments using CDX and PDX models with differing levels of CDH17 expression (H‐Score ranging from 20 to 230). A single dose of both AMT‐676 and AMT‐676‐E demonstrated significant anti‐tumor effects across multiple models, including GC CDX (Figure 2L), PDAC CDX (Figure 2M), PDAC PDX (Figure 2N) and BTC PDX models (Figure 2O,P). Remarkably, despite AMT‐676 having only half the DAR of AMT‐676‐E, it exhibited comparable activity in four out of five tested models. The BTC_PDX2 model revealed differential potency: while AMT‐676‐E induced tumor regression at both 3 and 10 mg kg^−1^, AMT‐676 achieved regression only at the higher dose (10 mg kg^−1^). These collective findings demonstrate that AMT‐676 maintains robust anti‐tumor activity comparable to AMT‐676‐E in the majority of experimental models.

**FIGURE 3:**
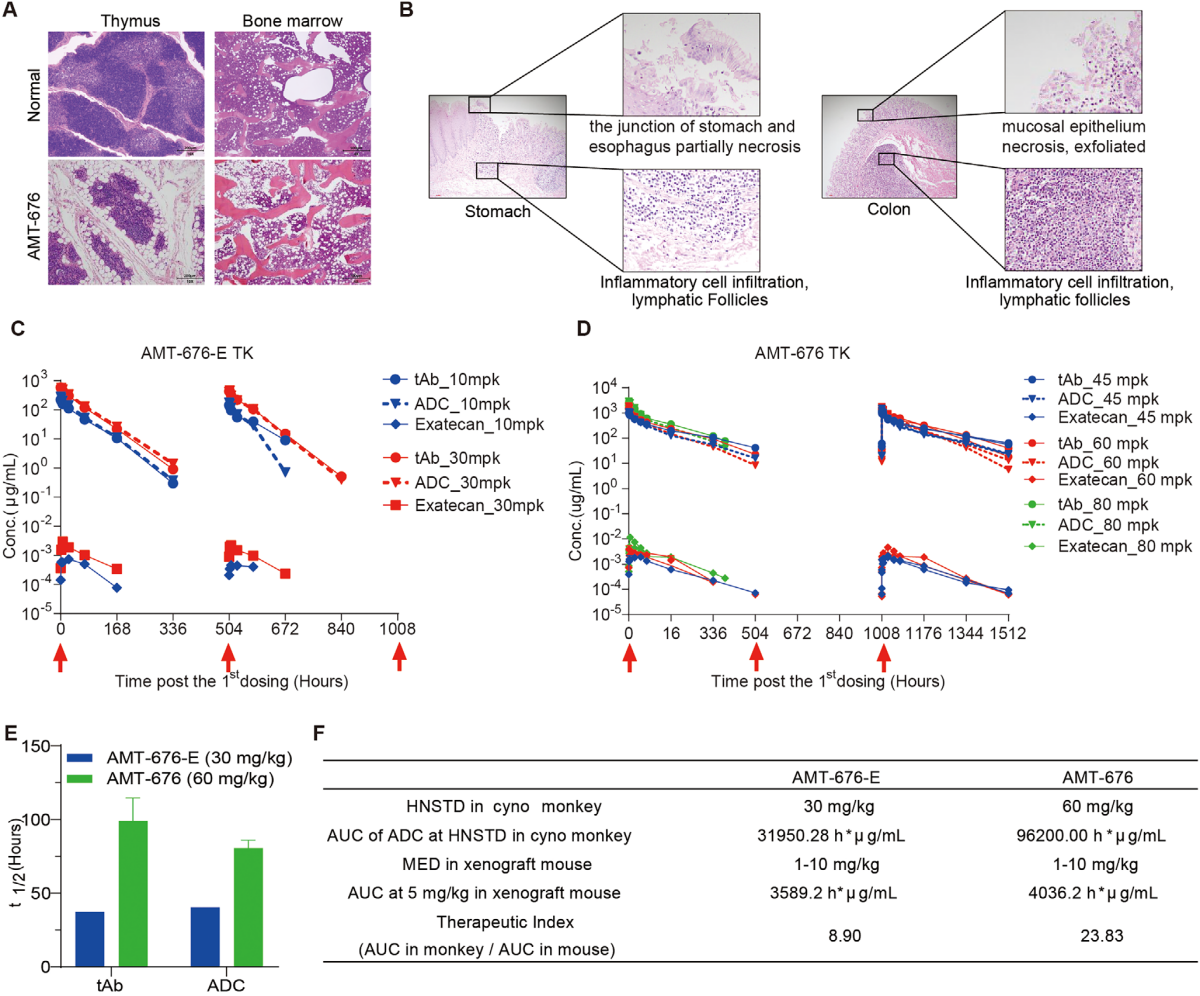
Toxicity assessment of CDH17‐targeting ADCs. (A) Representative H&E staining images illustrating toxic effects on target organs following administration of AMT‐676 in cynomolgus monkeys. (B) Representative H&E staining images depicting toxic effects on target organs after treatment with AMT‐676‐E. (C,D) Pharmacokinetic profiles for AMT‐676 (C) and AMT‐676‐E (D) in cynomolgus monkeys. (E) Half‐life determination of AMT‐676 and AMT‐676‐E at the highest non‐severely toxic dose (HNSTD) derived from repeated‐dose toxicity studies in cynomolgus monkeys. (F) Therapeutic index (TI) of AMT‐676 and AMT‐676‐E. TI was calculated by dividing the ADC exposure (AUC) associated with the highest non‐severely toxic dose (HNSTD) in monkey with that associated with minimum efficacious dose (MED) in xenograft mouse as previously reported [30, 31]. Data are represented as mean ± SD (E). Statistical analyses were performed using Student's t‐test (E). **, *p* < 0.01; ***, *p* < 0.001.

Next, we evaluated the toxicity and pharmacokinetics of AMT‐676 and AMT‐676‐E in cynomolgus monkeys in the pilot toxicology study, a species with a similar CDH17 expression pattern (Figure S2B). The animals received intravenous doses of AMT‐676 at 45, 60, and 80 mg kg^−1^, and AMT‐676‐E at 10 or 30 mg kg^−1^, administered every three weeks over three cycles. A single dose of AMT‐676 at 80 mg kg^−1^ led to early euthanasia of both animals, primarily due to gastrointestinal toxicities such as diarrhea, dehydration, decreased body weight and food intake, reduced motor activity, and hypothermia. Considering that GI toxicity is commonly observed with ADC using TOP1i payload and the fact that CDH17 is present in the normal intestines of both human and monkey, the GI adverse events observed at 80 mg kg^−1^ dose level were considered to be caused by, at least in part, on‐target toxicity. In contrast, repeated dosing of AMT‐676 at 45 and 60 mg kg^−1^ was well tolerated via intravenous infusion, with the highest non‐severely toxic dose (HNSTD) determined to be 60 mg kg^−1^ in the pilot toxicology study. Microscopic examination revealed changes limited to hemato‐lymphoid organs (bone marrow and thymus) at this HNSTD dose, with no evidence of on‐target toxicity in the target‐positive small and large intestines (Figure 3A). Conversely, AMT‐676‐E at its HNSTD dose of 30 mg/kg already caused pathological damage not only in hemato‐lymphoid organs but also in the GI tract (specifically the large intestine) (Figure 3B), suggesting a higher risk of potential on‐target toxicity with the DAR8 construct.

Overall, the major toxicities associated with both AMT‐676 and AMT‐676‐E were consistent with those observed in other topoisomerase I inhibitor‐based ADCs; however, the clinical and histopathological effects of AMT‐676‐E were more severe than those of AMT‐676 at their respective HNSTD doses (Table 1 and Figures S4 and S5). We further investigated the toxicokinetic profiles of both ADCs, detailing concentrations of AMT‐676, AMT‐676‐E, total antibody, and the released free payload exatecan in Figure 3C,D, with summary data provided in Tables S3 and S4. The exposure levels of both ADCs and total antibody increased proportionally with dose, and no drug accumulation was observed following repeated administration. Notably, AMT‐676 exhibited a longer average half‐life (T_1/2_) of 80.55 h compared to AMT‐676‐E, which had a T_1/2_ of 40.35 h at their respective HNSTD dose levels (Figure 3E; Tables S3 and S4).

**TABLE 1:** Summary of non‐GLP toxicity study in cynomolgus monkeys.

Species	Cynomolgus Monkeys	
Regimens	AMT‐676 ‐ E 0, 10, and 30 mg kg^−1^ Intravenous, every 3 weeks Days 1, 22, 43 (3 times in total)	AMT‐676 45, 60, and 80 mg kg^−1^ Intravenous, every 3 weeks Days 1, 22, 43 (3 times in total)
No. of animals	1/sex/group	1/sex/group
Lethal Dose	>30 mg kg^−1^	80 mg kg^−1^, euthanasia on Day 7 and Day 17
Clinical Signs	30 mg kg^−1^: Soft or liquid feces, occasional bloody feces or vomitus	≥45 mg kg^−1^: Fluid feces ≥60 mg kg^−1^: Dehydration, dry skin 80 mg kg^−1^: Eye(s) sunken, motor activity decreased, thin, hypothermia, prostrate
Food Intake	30 mg kg^−1^: Appetite reduction was noted	
Body Weight	30 mg kg^−1^: Upto ‐19.23% in female and ‐11% in male	60 mg kg^−1^: Upto ‐13% in male 80 mg kg^−1^: Upto ‐20%/‐12% in male/female
Hematology	30 mg kg^−1^: RET, RBC↓	≥45 mg kg^−1^: RET, RBC, HGB, HCT↓ ≥60 mg kg^−1^: WBC, NEUT, LYMP↓
Serum Chemistry	≤30 mg kg^−1^: normal	≤60 mg kg^−1^: normal 80 mg kg^−1^: UREA↑, CREA↑, Na↓, K↑, Cl↓
Target Organs and Tissues	≥10 mg kg^−1^: Brain, GI System 30 mg kg^−1^: Thymus	≥45 mg kg^−1^: Thymus ≥60 mg kg^−1^: Bone Marrow 80 mg kg^−1^: GI tract, Spleen, Adrenal Gland, Lymph Node, Skin, Lung
HNSTD	30 mg kg^−1^	60 mg kg^−1^

Abbreviations: RET, Reticulocyte; RBC, Red blood cell; HGB, Hemoglobin; HCT, Hematocrit; WBC, White blood cell; NEUT, Neutrophil; LYMP: Lymphocyte; UREA, Urea; CREA, Creatinine; NAD, No abnormality detected.

Furthermore, we calculated the therapeutic indices of AMT‐676 and AMT‐676‐E based on their corresponding HNSTD in monkeys and minimal effective dose (MED) in mice. Overall, AMT‐676 demonstrated a superior therapeutic index compared to AMT‐676‐E, thereby confirming its potential as the optimal candidate for CDH17‐targeting ADC therapy (Figure 3F).

### 2.3 The Molecular Mechanism of AMT‐676's Cytotoxic Effects

**FIGURE 4:**
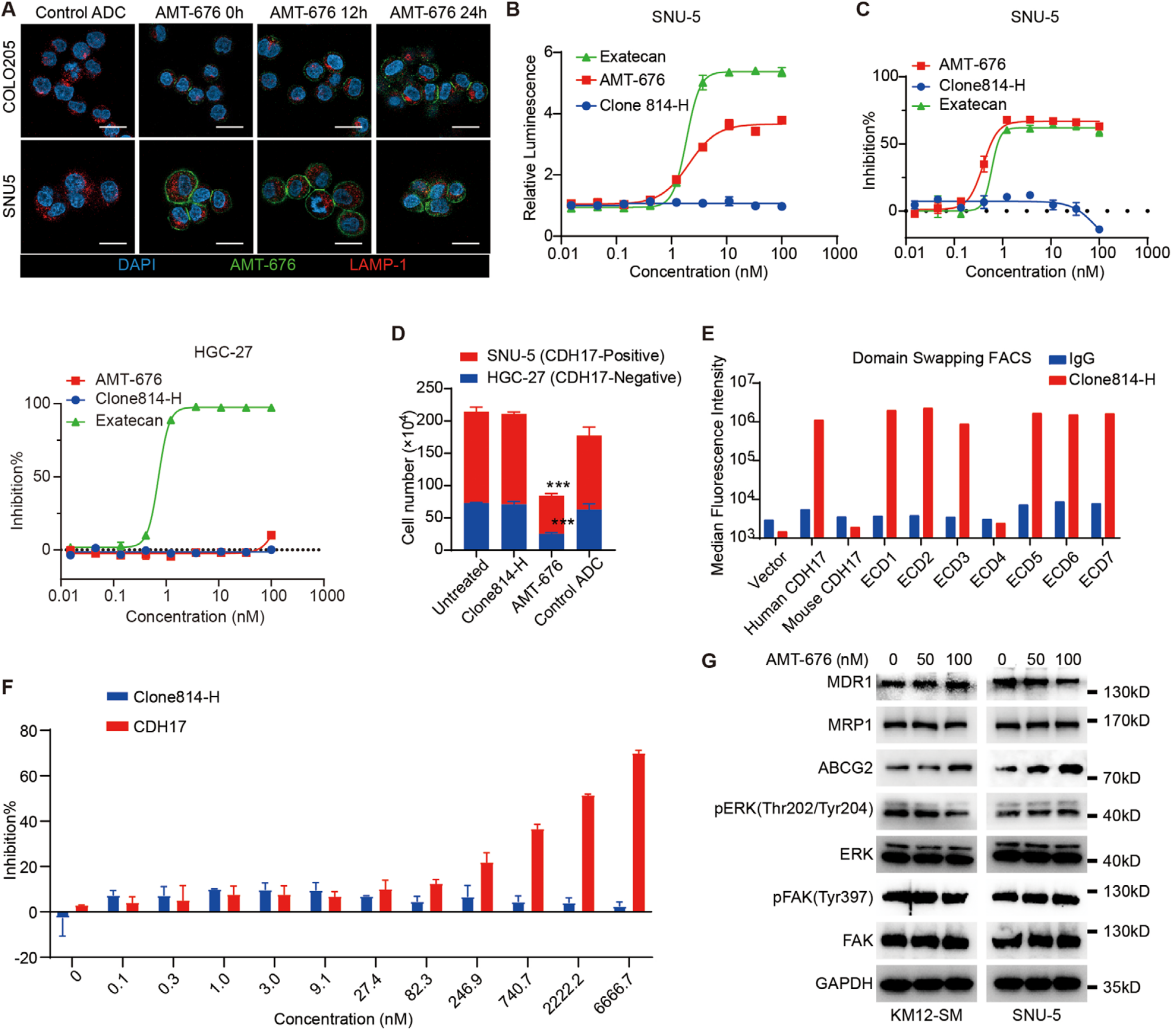
Molecular mechanisms of AMT‐676‐induced cytotoxicity. (A) COLO205 and SNU5 cells were treated with control ADC or AMT‐676. Colocalization analysis demonstrated the overlap between AMT‐676 (green) and the lysosome marker LAMP‐1 (red), indicating internalization of the ADC. (B) Evaluation of apoptosis induced by AMT‐676. SNU‐5 cells were treated with 10 nM of AMT‐676 and clone 814‐H for 48 h or with Exatecan for 24 h. The induction of apoptosis was measured through Caspase 3/7 activity using the Caspase‐Glo 3/7 assay (Promega, G8093) following the manufacturer's instructions. (C) In vitro cytotoxicity of AMT‐676, clone 814‐H, and Exatecan on SNU‐5 and HGC‐27 cells. (D) Assessment of the bystander killing effect of AMT‐676 under coculture conditions in vitro. SNU‐5 and HGC‐27 cells were cocultured and treated with 30 nM of ADCs for 5 days. Following treatment, adherent cells were collected, and the total cell count along with the ratio of CDH17‐positive to CDH17‐negative cells was quantified using a cell counter and flow cytometry, respectively. (E) Domain‐swapping experiments identified clone 814‐H's epitope on CDH17. (F) The interaction between CDH17 and integrin α2β1 was detected by competition ELISA. (G) SNU‐5 and KM12‐SM cells were treated with AMT‐676 for 3 days. Then the cells were collected for western blot analysis. Data are represented as mean ± SD (B‐D, F). Statistical analyses were performed using one‐way ANOVA (D). ***, *p* < 0.001.

ADCs exert their cytotoxic effects through endocytosis, followed by the release of therapeutic agents within target cells, resulting in cell death. To evaluate the internalization of AMT‐676, we treated the CRC cell line COLO205 and the GC cell line SNU‐5, both of which express CDH17, with control ADC or AMT‐676. Confocal microscopy confirmed the subcellular localization of AMT‐676 to lysosomes, revealing a time‐dependent increase in its internalization (Figure 4A). Additionally, treatment of SNU‐5 cells with either AMT‐676 or the free payload exatecan increased caspase 3/7 apoptotic signals (Figure 4B), consistent with the mechanism of a TOP1‐inhibitor payload. Exatecan‐based ADCs have been shown to have strong bystander activity [25]. We assessed the bystander‐killing effect of AMT‐676 using CDH17‐positive SNU‐5 cells and CDH17‐negative HGC‐27 cells in an in vitro co‐culture assay. We first confirmed that AMT‐676 could effectively induce cell death in CDH17‐positive SNU‐5 cells but showed no activity against CDH17‐negative HGC‐27 cells, whereas the free payload exatecan potently killed both cell lines (Figure 4C). Co‐culture experiments demonstrated that AMT‐676 killed both CDH17‐positive SNU‐5 cells and CDH17‐negative HGC‐27 cells, whereas the unconjugated antibody clone 814‐H or the isotype control ADC showed no activity against either cell line (Figure 4D). These results confirmed AMT‐676's bystander‐killing effect compared to the negative control ADC.

Previous studies have demonstrated that the RGD motif within the sixth extracellular domain (ECD6) of CDH17 can interact with integrins to activate downstream signaling pathways [32]. To determine whether AMT‐676 inhibits tumors through this specific mechanism, we first mapped the binding epitope of the clone 814‐H antibody on CDH17 and identified it as ECD4 (Figure 4E). Based on this finding, we hypothesized that AMT‐676 would not interfere with the activation of integrin‐mediated signaling pathways. To experimentally validate this hypothesis, we performed competition ELISA to evaluate the integrity of the CDH17–α2β1 integrin interaction and Western blot analyses to examine the activation status of downstream signaling cascades. The results consistently showed that AMT‐676 binding to CDH17 neither disrupts the CDH17–α2β1 interaction nor modulates the activation of the associated signaling pathways (Figure 4F,G). In addition, we detected the expression of ABC transporters, including ABCG2, MRP1 and MDR1, following stimulation with AMT‐676. Our results showed a significant upregulation of ABCG2 levels upon exposure to AMT‐676, whereas MDR1 and MRP1 showed no substantial changes (Figure 4G). These findings suggest that ABCG2‐mediated efflux may contribute to the resistance by reducing intracellular drug accumulation, thereby diminishing the cytotoxic effects of AMT‐676.

### 2.4 AMT‐676 Suppresses CRC Metastasis In Vivo

**FIGURE 5:**
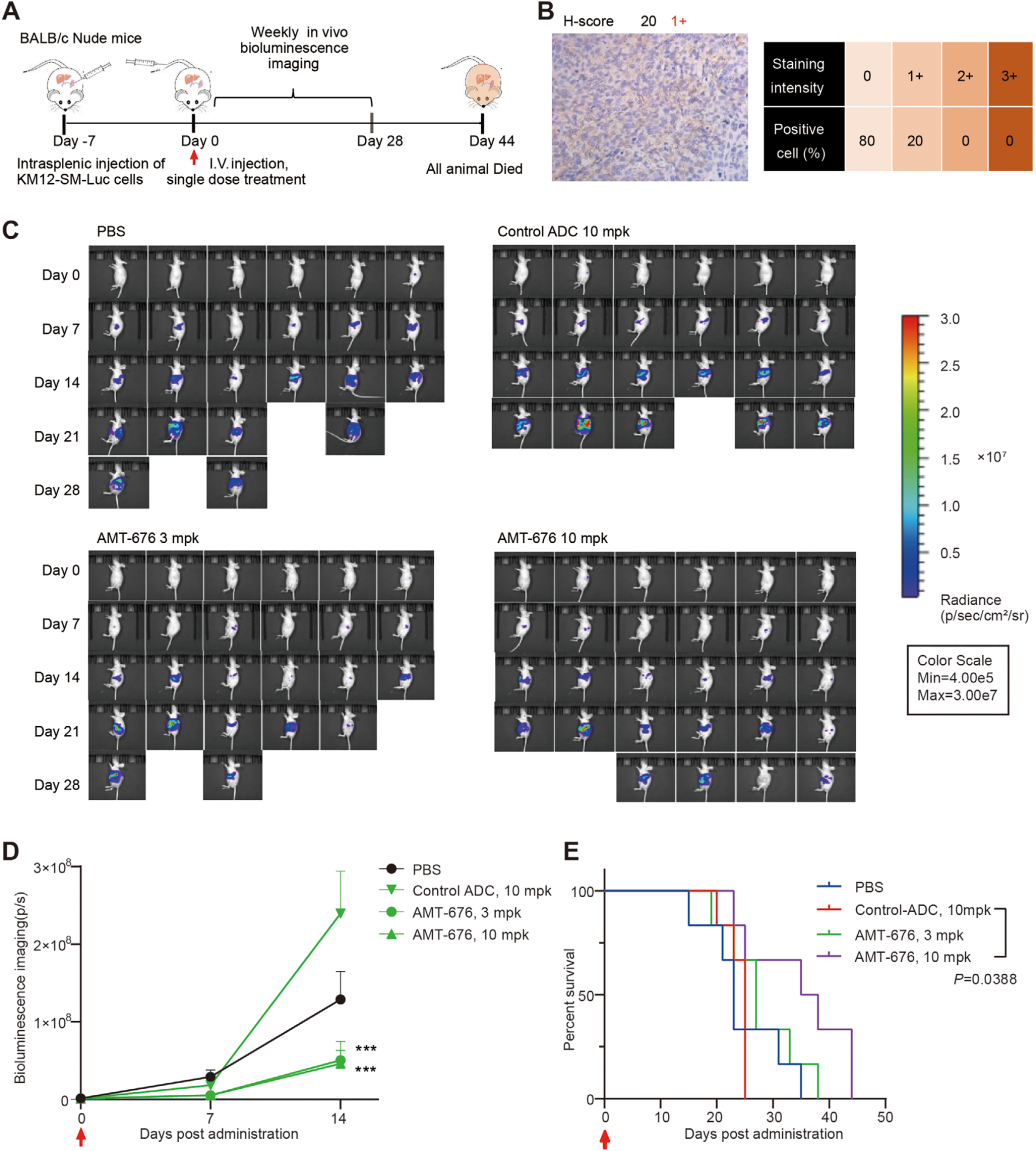
AMT‐676 inhibits liver metastasis in CRC. (A) Experimental scheme of the KM12‐SM‐Luc liver metastatic xenograft model (*n* = 6). (B) Assessment of CDH17 expression in the KM12‐SM‐Luc liver metastatic xenograft model using IHC. (C) In vivo whole‐body bioluminescence imaging of anesthetized mice treated with a single intravenous dose of vehicle, isotype control ADC, or AMT‐676 at indicated dose levels, conducted from day 0 to day 28 to illustrate tumor progression over time. (D) Quantification and analysis of bioluminescence signals from liver metastases. Statistical comparisons were performed between AMT‐676 3 mpk, 10 mpk and control ADC (Ritu‐T1000‐Exatecan(DAR4)), respectively. (E) Kaplan‐Meier survival curves of KM12‐SM‐Luc tumor‐bearing mice following treatment. Data are represented as mean ± SEM (D). Statistical analyses were performed using two‐way ANOVA (D) and Log‐rank test (E). *, *p* < 0.05; **, *p* < 0.01; ***, *p* < 0.001.

The liver is a common site for metastasis in CRC. To evaluate the efficacy of AMT‐676, we established a liver metastasis model using the highly metastatic KM12‐SM human CRC cell line [33] engineered to express luciferase (KM12‐SM‐Luc) in nude mice (Figure 5A). IHC confirmed the expression of CDH17 in the KM12‐SM cells (Figure 5B). The results demonstrated that AMT‐676 significantly suppressed the growth of liver metastases (Figure 5C,D). Furthermore, survival analysis indicated that treatment with AMT‐676 markedly prolonged the survival of mice with liver metastases, highlighting its potential as a therapeutic option for metastatic tumors (Figure 5E).

### 2.5 AMT‐676 Effectively Inhibits Growth of Many Types of GI Tumors

**FIGURE 6:**
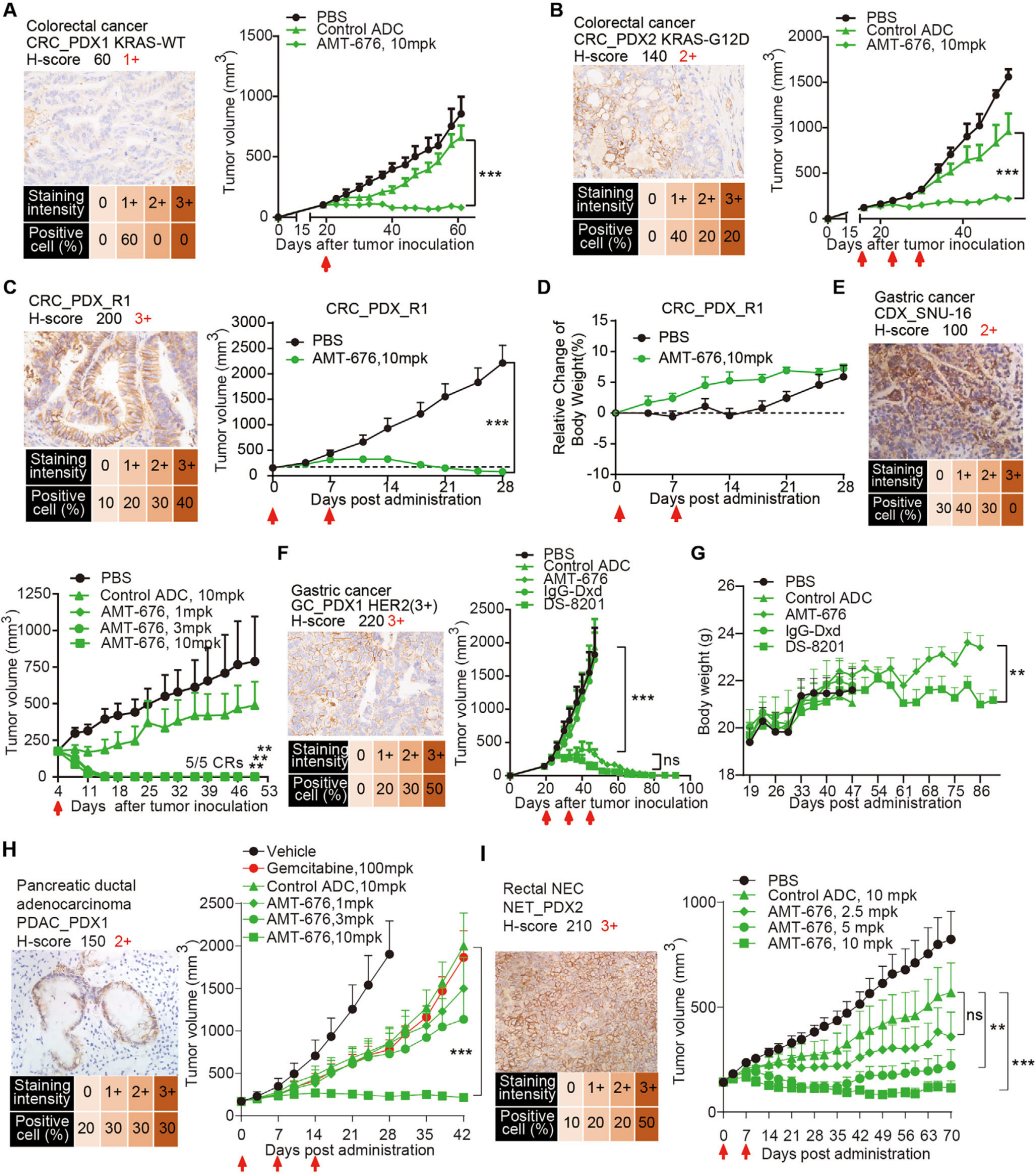
In vivo efficacy of AMT‐676 in CDH17‐expression gastrointestinal cancers. (A,B) Establishment of CRC PDX models (*n* = 5) in nude mice. Mice were administered with PBS, control ADC (Ritu‐T1000‐Exatecan (DAR4)), or AMT‐676. The left panel showed CDH17 expression in untreated mouse tumor tissue, as indicated by IHC images and the corresponding H‐scores. The right panel presented the tumor growth curves for each treatment group. (C,D) In vivo efficacy of AMT‐676 in a chemoresistant CRC model (*n* = 5). In (C), the CDH17 expression in untreated tissues and efficacy of AMT‐676 were presented. Meanwhile, (D) shows the percentage change in the body weight of the mice. (E) SNU‐16 GC cells were subcutaneously inoculated into nude mice (*n* = 5). Both the IHC staining results for CDH17 and the tumor growth curves were shown. Statistical comparisons were performed between AMT‐676 1 mpk, 3 mpk, 10 mpk and control ADC (Ritu‐T1000‐Exatecan(DAR4)), respectively. (F) GC PDX models (*n* = 5) treated with the PBS, control ADC (Ritu‐T1000‐Exatecan(DAR4)), AMT‐676, IgG‐Dxd (Ritu‐GGFG‐DXd(DAR8)), or DS‐8201. The IHC staining for CDH17 and the corresponding tumor growth curves are displayed. (G) The body weight of the mice in (F) was monitored every 3–4 days. Data are represented as mean ± SEM (A‐I). (H‐I) The efficacy of AMT‐676 was evaluated in PDAC PDX models (*n* = 5) and NET PDX models (*n* = 5) originating from the rectum. Statistical analyses were performed using two‐way ANOVA with Tukey's post hoc test (A‐I). *, *p* < 0.05; **, *p* < 0.01; ***, *p* < 0.001.

Given the widespread expression of CDH17 across different GI tumors, we investigated the therapeutic efficacy of AMT‐676 in CDX and PDX models of GI cancers (Figure 6). In CRC PDX models, AMT‐676 significantly inhibited tumor growth regardless of whether the KRAS gene was in its wild‐type (WT) state or carried the G12D mutation (Figure 6A,B). Drug resistance to frontline chemotherapy, including regimens with irinotecan, poses a substantial hurdle to CRC treatments. CRC_PDX_R1 (LD1‐0038‐361928) is a PDX model established from rectal cancer patients previously treated with Bevacizumab, Capecitabine, Cetuximab, Pyrotinib, FOLFOX regimen, XELOX regimen, and FOLFIRI regimen. The model exhibits resistance to the combination of irinotecan, calcium folinate, and 5‐fluorouracil. In this chemo‐resistant CRC PDX model, AMT‐676 demonstrated potent antitumor activity without causing weight loss in the mice, indicating its ability to overcome chemotherapy resistance in CRC (Figure 6C,D). Moreover, in the GC SNU‐16 CDX model, AMT‐676 effectively suppressed tumor growth at all three tested concentrations (1, 3, and 10 mg kg^−1^) (Figure 6E). Notably, at equivalent dosages, the therapeutic efficacy of AMT‐676 was comparable to that of DS‐8201, an approved ADC targeting HER2 (Figure 6F). Additionally, the mice treated with AMT‐676 experienced greater body weight gain compared to the DS‐8201 group, suggesting that AMT‐676 has fewer side effects (Figure 6G). Furthermore, AMT‐676 exerted an anti‐tumor effect in one PDAC PDX model and one NET PDX model originating from the rectum (Figure 6H,I). Overall, AMT‐676 demonstrated tumor regression or significant tumor inhibition in a diverse range of animal models representing CRC, GC, PDAC, and NETs.

## 3 Discussion

Despite significant advancements in the treatment of gastrointestinal (GI) cancers, substantial unmet medical needs remain. In this study, we developed AMT‐676, a novel antibody‐drug conjugate (ADC) targeting CDH17 in GI tumors. This ADC is based on the T moiety‐exatecan platform and comprises a high‐affinity, CDH17‐specific antibody, a hydrophilic self‐immolative T1000 linker, and exatecan, with a drug‐to‐antibody ratio (DAR) of 4. In vitro experiments demonstrated the efficacy of AMT‐676 in inducing cytotoxicity in tumor cell lines, along with a robust bystander effect. In vivo, AMT‐676 exhibited durable antitumor responses in both CDX and PDX models across varying levels of CDH17 expression, including in metastatic tumors. Despite its potent activity, AMT‐676 displayed a favorable safety profile in cynomolgus monkeys, indicating its potential as a promising ADC for the treatment of GI tumors.

CDH17 has been recognized as both a tumor promoter and a diagnostic marker in various GI tumors [14]. The suppression of CDH17 has been shown to activate apoptosis signaling pathways, thereby promoting apoptosis in colorectal and pancreatic cancer cells [34, 35]. Recent studies have highlighted the role of the RGD motif within the extracellular domains in activating integrin pathways, which contribute to malignant tumor progression [36]. Our analysis revealed CDH17 expression levels were significantly higher in PIK3CA‐mutant CRC tumors compared to PIK3CA wild‐type CRC tumors, suggesting that PIK3CA mutations may upregulate CDH17 expression (Figure 1B). Further investigation into the underlying mechanisms could provide valuable insights. In GC, we observed that a substantial proportion of HER2‐positive patients also express CDH17, indicating that this group may benefit from targeted CDH17 therapies (Figure 1C). While elevated CDH17 expression is traditionally associated with poor patient survival in colorectal cancer, GC, and hepatocellular carcinoma [37, 38, 39], our comprehensive analysis across GI tumors revealed varying prognostic correlations among different cancer types (Figure 1E). This heterogeneous association underscores the high tissue specificity and context dependence of CDH17 functions. Its dual role as an adhesion molecule and potential signaling molecule may be differentially regulated or utilized in diverse tumor microenvironments, leading to contrasting effects on tumor biology and patient outcomes. Furthermore, we identified distinct optimal H‐score cut‐off values for CDH17 levels in OS analysis, which were determined to be 217.5 in CRC, 8.5 in GC, 0.5 in PDAC, and 13 in BTC. Therefore, establishing CDH17 as a universal prognostic biomarker across gastrointestinal cancers is challenging, and its value must be evaluated strictly within the context of specific cancer types.

The critical role of CDH17 in GI cancers has spurred the development of various targeted therapies, including monoclonal antibodies, bispecific antibodies, chimeric antigen receptor (CAR) T‐cell therapy, and ADCs. Monoclonal antibodies targeting the RGD motif have demonstrated efficacy in preclinical models of CRC and melanoma [40, 41]. In addition, BI 905711, a novel tetravalent bispecific antibody that targets both TRAILR2 and CDH17, has shown potent antitumor activity in CDH17‐positive CRC models [42]. Another bispecific antibody, ARB201, has also exhibited antitumor effects in preclinical studies through its ability to bind both CD3‐positive T cells and CDH17‐positive tumor cells [43]. Furthermore, CDH17‐targeting CAR‐T cells have demonstrated strong cytotoxic activity against CDH17‐positive NETs and various GI cancers, including small cell lung cancer (SCLC), GC, PDAC, and CRC [24, 27]. These promising bispecific antibodies and CAR‐T therapies have entered early‐stage clinical trials (NCT04137289, NCT06501183, NCT06055439).

ADCs represent another promising drug modality, with some having received approval for use in GI cancers such as gastric and colorectal cancer [7, 44, 45, 46]. CDH17, a target of interest in these cancers, is predominantly localized to the basolateral membrane domain of healthy polarized epithelial cells, positioned beneath apical tight junctions [24]. This anatomical restriction forms a critical physical and functional barrier that limits ADC access from the luminal side, a mechanism thought to mitigate off‐target binding and toxicity in normal CDH17‐expressing tissues. Against this backdrop, CDH17‐targeting ADCs such as TORL‐3‐600 and ARB102A, both utilizing monomethyl auristatin E (MMAE) as the cytotoxic payload, have demonstrated anti‐tumor activity in CDH17‐positive colorectal cancer (CRC) and pancreatic cancer models, with TORL‐3‐600 currently under evaluation in a Phase I clinical trial (NCT05948826) [47, 48]. However, MMAE‐based ADCs are known to possess a narrow therapeutic window, which may not be ideal for targets like CDH17, given its moderate to high expression in normal intestinal tissues. In contrast, AMT‐676 was developed based on the T moiety‐exatecan platform. Previous studies indicated that an ADC incorporating the T moiety‐exatecan demonstrated a higher therapeutic index compared to a counterpart ADC using the same antibody conjugated with MMAE. In addition, exatecan is less susceptible than MMAE or some other TOP1 inhibitor payloads to multidrug resistance mechanisms such as Pgp/ABCG2 efflux pumps, which are common features of GI cancers. To further enhance the therapeutic index and mitigate potential on‐target toxicity to CDH17‐expressing normal intestine, we optimized the drug‐to‐antibody ratio (DAR) of the ADC by comprehensively evaluating the in vivo anti‐tumor efficacy and safety profiles of two designs: DAR4 and DAR8. A notable finding in this study is the striking difference in pharmacokinetic (PK) behavior between the DAR4 and DAR8 variants in cynomolgus monkeys, whereas in mice, the two variants exhibited nearly indistinguishable PK profiles. This species discrepancy is mechanistically informative. Our anti‐CDH17 antibody specifically recognizes human and cynomolgus monkey CDH17, but does not cross‐react with mouse CDH17. Consequently, in mice, there is no target‐mediated drug disposition (TMDD). In cynomolgus monkeys, however, the antibody binds to endogenous CDH17 expressed on normal tissues, particularly along the gastrointestinal tract. This binding introduces TMDD, which is known to accelerate clearance. Our observation that DAR8 exhibits significantly different PK (e.g., faster clearance, lower exposure) compared to DAR4 in monkeys suggests that the more potent DAR8 construct may disrupt the lateral localization of CDH17 — a feature that is a prerequisite for reducing the on‐target off‐tumor toxicity of CDH17 ADCs. These findings again underscore the importance of DAR optimization for targets like CDH17, which exhibit different cell surface exposure patterns between tumor and normal tissue. Consequently, AMT‐676, the DAR4 ADC, achieved a higher therapeutic index due to comparable or only slightly reduced efficacy while exhibiting significantly better tolerance at higher dose levels. Regarding the observed toxicity, we propose that it reflects an additive or synergistic interplay between on‐target component and off‐target/platform‐related components. Mechanistically, at very high systemic concentrations, the ADC may overcome the basolateral barrier, binding to CDH17 on normal intestinal epithelial cells and delivering the payload, thereby inducing cellular damage. Concurrently, gastrointestinal epithelial toxicity is a known class effect of TOP1i‐based ADC platforms, further contributing to this phenotype. Additionally, AMT‐676 possesses a robust bystander effect in vitro, which may contribute to its strong antitumor activity even in CDH17‐low models. Notably, the isotype control ADC also exhibited partial anti‐tumor activity in certain contexts, such as the IHC 1+ model (Figure 2N). This baseline effect likely stems from target‐independent ADC uptake, extracellular cleavage within the tumor microenvironment, or trace non‐specific payload release. Nevertheless, AMT‐676 maintained substantially superior efficacy, confirming its target‐dependent specificity. In vivo, AMT‐676 induced durable antitumor responses in CDX and PDX models representing a wide range of GI tumors with varying CDH17 expression levels, highlighting its therapeutic potential.

Metastasis remains the primary cause of mortality in GI cancers. Studies indicate that CDH17 expression is often well‐preserved during the metastatic process [49]. Indeed, CDH17 serves as a sensitive biomarker for detecting metastases of unknown primary origin, aiding in their subsequent classification as originating from the GI tract [50]. Research has shown that dysregulated cadherin expression can promote invasion and metastasis in various cancers [51]. Specifically, CDH17 has been identified to directly bind and activate α2β1 integrin through its RGD motifs, facilitating cell adhesion and tumor metastasis [36, 52, 53]. In our liver metastasis model, AMT‐676 demonstrated significant inhibition of tumor growth and prolonged survival in mice (Figure 5). These findings provide critical preclinical evidence supporting the potential application of AMT‐676 in the treatment of metastatic GI tumors.

In summary, we have successfully developed AMT‐676, an exatecan‐based ADC targeting CDH17, which exhibits a high therapeutic index for CDH17‐positive GI tumors. A first‐in‐human Phase I study evaluating AMT‐676 in patients with advanced solid tumors is currently underway (NCT06400485).

## 4 Methods

### 4.1 Patients and Tissue Samples

In this study, we obtained formalin‐fixed, paraffin‐embedded (FFPE) from patients diagnosed with CRC (*n* = 80), EAC (*n* = 31), GC (*n* = 94), PDAC (*n* = 85), BTC (*n* = 31), NET (*n* = 66) tissues at Sun Yat‐sen University Cancer Center (Guangzhou, China) between January 2015 and December 2020. Histopathological confirmation of the diagnosis was achieved for all patients. Additionally, high‐throughput sequencing data from the CRC cohort and HER2 IHC scores from the GC samples were collected. Informed consent was obtained from each participant, and the study received approval from the Institutional Review Board of Sun Yat‐sen University Cancer Center (license number B2023‐477).

### 4.2 Antibodies, ADCs and Compounds

The anti‐CDH17 humanized antibody (clone 814‐H) was generated using a CHO expression system and purified with MabSelect Sure Resin (GE Healthcare Life Sciences) via gravity columns. Isotype IgG was obtained from Beijing Solarbio Science & Technology Co., Ltd. ADCs were synthesized as described below, and their DAR values were determined using reverse‐phase liquid chromatography (RPLC). Drug distribution was analyzed through HIC. AMT‐676 and AMT‐676‐E were produced in‐house using linker‐payloads synthesized as previously reported [25]. The endotoxin levels of AMT‐676 and AMT‐676‐E were found to be less than 0.05 EU/mg.

### 4.3 Cell Lines and PDX Models

The COLO205 cell line (RRID: CVCL_0218) was purchased from the American Type Culture Collection (ATCC) in 2020. The KM12‐SM (RRID: CVCL_9548), SNU‐5 (RRID: CVCL_0078), SNU‐16 (RRID: CVCL_0076), HGC‐27 (RRID: CVCL_1279), and AsPC‐1 (RRID: CVCL_0152) cell lines were acquired from MeisenCTCC (Zhejiang, China) in 2021 and 2023. The PDX models in Figure 6A–C were obtained from Sun Yat‐sen University Cancer Center. All other PDX models were sourced from Lidebiotech Co., Ltd. Cells were maintained in RPMI‐1640 medium (for COLO205, SNU‐5, SNU‐16, and AsPC‐1) or DMEM (for KM12‐SM and HGC‐27), supplemented with 10% heat‐inactivated fetal bovine serum (FBS), and cultured at 37°C in a 5% CO_2_ atmosphere. When cells reached approximately 80% confluence, they were dissociated from culture plates using 1× PBS and 1 mM EDTA for 10 to 20 min. All cell lines were authenticated through short tandem repeat (STR) analysis according to supplier information. Prior to conducting cell‐based assays, all cells tested negative for Mycoplasma contamination using a Mycoplasma PCR Detection Kit (GeneCopoeia, Rockville, MD, USA, MP004). All cells and PDX models were utilized for experiments within 5 to 15 passages and 2 to 5 passages, respectively, following collection.

### 4.4 Immunofluorescence and Flow Cytometry

Immunofluorescence (IF) and flow cytometry assays were conducted under non‐permeable conditions. Antibody binding signals were detected using Alexa Fluor 488 and 594 goat anti‐mouse IgG secondary antibodies (Jackson ImmunoResearch Labs, Cat# 115‐545‐003, RRID:AB_2338840 and Cat# 115‐585‐003, RRID:AB_2338871), or Alexa Fluor 488 goat anti‐human IgG secondary antibody (Jackson ImmunoResearch Labs Cat# 109‐545‐008, RRID:AB_2337833).

In brief, cells adhered to coverslips (for IF assays) or suspended in 1× PBS (for flow cytometry assays) were first fixed in 4% paraformaldehyde (PFA) for 10 min. Following fixation, PFA was removed, and cells were rinsed three times with 1× PBS. Cells were then blocked overnight at 4°C in a blocking buffer consisting of 1× PBS containing 10% normal goat serum. After removing the blocking buffer, cells were incubated with the primary antibody (diluted in the blocking buffer at a ratio ranging from 1:100 to 1:1000 according to the manufacturer's instructions) for 2 h at room temperature (RT). Following three rinses with 1× PBS, cells were incubated with the fluorescence‐labeled secondary antibody (diluted in blocking buffer at a 1:500 dilution along with 1:10 000 Hoechst 33258, Sigma–Aldrich, St. Louis, MO, USA) for 1 h. Finally, cells were rinsed three times with 1× PBS.

IF images were captured using an Olympus CKX53 fluorescence microscope. Merging of the IF results was performed using ImageJ (ImageJ, RRID:SCR_003070). Flow cytometry data were collected with a BD Accuri C6 Plus system. Control samples without primary antibody and those with isotype control antibodies were included for comparison. The LIVE/DEAD Fixable Near‐IR Dead Cell Stain Kit (Thermo Fisher Scientific, Waltham, MA, USA, L34960) was utilized to exclude dead cells from analysis. Relative mean fluorescence intensity (rMFI) was calculated as the ratio of the MFI of the anti‐target antibody‐FITC to the MFI of the isotype control‐FITC.

### 4.5 Binding Domain Mapping

The binding domain of clone 814‐H on human CDH17 was determined by a domain swapping approach. To construct domain swapping plasmids, each of the seven extracellular domains (ECD1, ECD2, ECD3, ECD4, ECD5, ECD6, and ECD7) of human CDH17 were replaced with the corresponding extracellular domain sequences of mouse CDH17, and the constructs were also fused with GFP protein, resulting in pcDNA3.1‐ECD1‐GFP, pcDNA3.1‐ECD2‐GFP, pcDNA3.1‐ECD3‐GFP, pcDNA3.1‐ECD4‐GFP, pcDNA3.1‐ECD5‐GFP, pcDNA3.1‐ECD6‐GFP and pcDNA3.1‐ECD7‐GFP, respectively. The plasmids were then transfected into 293F following standard procedure. Then, cells were harvested by centrifugation at 1000 rpm for 5 min. The cells were then subjected to flow cytometry following the procedure described above using clone 814‐H as the primary antibody.

### 4.6 Immunohistochemistry

After deparaffinization and rehydration, formalin‐fixed, paraffin‐embedded (FFPE) sections were treated with antigen retrieval buffer (DAKO) and incubated with the anti‐CDH17 antibody clone 814 or anti‐CLDN18.2 antibody (Abcam, ab241330, RRID: AB_3675979) for 1 h. Following this incubation, the sections were washed and then incubated with an HRP‐labeled secondary antibody (DAKO) for an additional hour. Target expression was detected using DAB staining buffer and hematoxylin (Harris). The histochemical scoring system (H‐score) was employed to evaluate the intensity of target expression. The expression levels were classified as follows: 0 (H‐score 0–9), 1+ (H‐score 10–99), 2+ (H‐score 100–199), and 3+ (H‐score 200–300).

### 4.7 Indirect Cytotoxicity Assay for ADC

In the indirect cytotoxicity assay, a strategy utilizing a vc‐MMAE conjugated anti‐mouse IgG antibody was employed as a surrogate for directly conjugating mAbs, enabling the screening of mAb candidates, as previously described [54]. To assess endocytosis‐mediated cytotoxicity, cancer cells were plated at a density of 2000 cells per well in a 96‐well plate and cultured overnight. Following this incubation, cells were treated with 1 nM of mAb in conjunction with 2 µg mL^−1^ MMAE‐conjugated secondary goat anti‐mouse IgG antibody for 72 h. Cell viability was subsequently assessed using the CCK‐8 assay (Dojindo, Kumamoto, Japan, #CK04‐20).

### 4.8 Antibody Humanization

Clone 814 was humanized using a CDR‐grafting approach following a previously described method [29]. Briefly, the sequence of clone 814 was analyzed using IMGT domains [55, 56]. The CDRs of the heavy chain and light chain were grafted to human germline sequences IGHV1‐46*01 and IGKV1‐NL1*01, respectively. The humanized antibody was expressed, and its binding affinity was evaluated by flow cytometry on CDH17‐positive Colo205 cells.

### 4.9 ADC Preparation

The preparation of the T moiety‐exatecan ADC was performed as follows. A solution of naked mAb was prepared at 10 mg/mL concentration in PBS (pH 7.0) supplemented with 2 mM EDTA. Then the mAb solution was treated with 7 molar equivalents of tris(2‐carboxyethyl)phosphine (TCEP) for AMT‐676, or 2.5 molar equivalents for AMT‐676‐E, and incubated for 2 h at 37°C. Subsequently, 14 (for AMT‐676) or 7 (for AMT‐676‐E) molar equivalents of linker‐drug (from a 10 mM DMA stock solution) were added to the reduced antibody while ensuring that the residual DMA concentration remained below 10% (v/v). This mixture was incubated at room temperature for one hour. Purification was then carried out using a Zeba spin desalting column (Thermo Fisher Scientific) to remove excess reagents. During this step, the resulting ADC was also buffer exchanged into a formulation buffer consisting of 20 mM histidine and 150 mM NaCl at pH 5.5. To ensure thorough removal of any excess linker‐drug, the product was treated with activated charcoal (30 mg of charcoal per 1 mL of ADC solution) and vortexed for 2 h at room temperature. The charcoal was subsequently removed via sterile filtration using 0.2 µmµ PES filters, and the final ADC was stored at −80°C.The concentration, drug‐to‐antibody ratio (DAR), and aggregation of the antibody‐drug conjugate were measured by UV absorbance, HIC, and SEC, respectively, as previously reported [25].

A DS‐8201 analog was prepared according to the conjugation procedure reported previously [57]. Isotype control ADCs for AMT‐676, AMT‐676‐E, and DS‐8201 were prepared by conjugating each corresponding linker‐payload to a non‐binding human IgG to achieve the respective DAR.

### 4.10 Bystander Killing Effect

The bystander killing effect of AMT‐676 and isotype control IgG‐T1000‐exatecan ADC was evaluated using an in vitro co‐culture system consisting of CDH17‐positive SNU‐5 cells and CDH17‐negative HGC‐27 cells. Following five days of incubation with the respective treatments in 6‐well plates, adherent cells were harvested. The total cell count and the ratio of CDH17‐positive to CDH17‐negative cells were assessed using a cell counter (Countess3, Invitrogen) and a flow cytometer (BD Accuri C6 Plus), respectively.

### 4.11 Internalization Assay

The internalization of isotype control ADC and AMT‐676 by CDH17‐positive COLO205 and SNU‐5 cells was assessed. The cells were treated with AMT‐676 and isotype control ADC for 12 and 24 h. Following treatment, the cells were washed twice with PBS and fixed in 4% paraformaldehyde for 15 min. After two additional washes with PBS, the cell membranes were permeabilized using a 0.2% Triton X‐100 solution for 10 min. Subsequently, the cells were washed twice with PBS and incubated overnight with a primary antibody against Human LAMP‐1 (CST, Danvers, MA, USA, #15665). The cells were then incubated at RT for 1 h with AF594‐Goat anti‐Mouse IgG (H+L) (CST, #8890) and FITC‐Goat Anti‐Human IgG (H+L) (Abcam, Cambridge, MA, USA, ab6854). Finally, the cells were stained with DAPI, and images were acquired using a confocal microscope.

### 4.12 Western Blot Analysis

KM12‐SM and SNU‐5 cells were treated with AMT‐676 or vehicle control for 72 h. Post‐treatment, cells were harvested and lysed in RIPA buffer supplemented with protease and phosphatase inhibitor cocktail. Protein samples underwent SDS‐PAGE separation and were subsequently transferred to PVDF membranes. Membranes were blocked with 5% BSA followed by overnight incubation at 4°C with primary antibodies. After washing, membranes were incubated with HRP‐conjugated secondary antibodies, and protein signals were visualized using enhanced chemiluminescence (ECL) substrate. Primary antibodies used: GAPDH (Cell signaling technology, Danvers, MA, USA; #2118, 1:1000), FAK (CST #3285, 1:1000), pFAK(Tyr397) (CST #3283, 1:1000), ERK (CST #4695, 1:1000), pERK(Thr202/Tyr204) (CST #4730, 1:1000), ABCG2 (Immunoway, Suzhou, China; YT0053, 1:1000), MRP1 (Immunoway, YN0885, 1:1000), MDR1 (Immunoway, YM8353, 1:1000).

### 4.13 Animal Models

AsPC‐1 and SNU‐16 CDX models were established in female BALB/c Nude mice, while the SNU‐5 CDX was developed in female CB‐17 SCID mice. The NET_PDX1 model was established in female NCG mice, and all other PDX models were generated in female nude mice. All in vivo studies were conducted following the applicable regulations and local guidelines of the Institutional Animal Care and Use Committee.

Briefly, 4 to 6‐week‐old mice were housed collectively in sterilized cages and maintained under pathogen‐free conditions. Each cell suspension or tumor fragment was inoculated subcutaneously into the respective mice. Once the tumors reached an appropriate size, tumor‐bearing mice were randomized into treatment and control groups based on tumor volume, and treatment was initiated. Antibodies and ADCs were administered intravenously, whereas small molecule drugs were given orally. Tumor volume defined as 1/2 × length × width^2^ was measured twice a week (every 3–4 days). Mice were euthanized using CO_2_ gas when they reached predetermined endpoints, including a tumor volume exceeding 3000 mm^3^, greater than 20% body weight reduction, or any clinical signs indicating the need for euthanasia for ethical reasons.

To study the effect of AMT‐676 on CRC liver metastasis in mice, KM12‐SM‐Luc cells suspended in 100 µL PBS were inoculated with a surgical injection into the spleen of BALB/c nude mice. 7 days after inoculation, all the mice were imaged by IVIS to confirm if the luminescence was visible. The animals with luminescence were grouped into 4 groups by MFI and body weight and received a single dose of AMT‐676. Mice were imaged in Day 0, Day 7, Day 14, Day 21, and Day 28 after grouping and were monitored for survival.

### 4.14 Pharmacokinetics of AMT‐676 and AMT‐676‐E in Mice

AMT‐676 and AMT‐676‐E were intravenously administered at a dosage of 5 mg kg^−1^ to SNU‐5 tumor‐bearing mice. Plasma concentrations of the ADC and total antibody were measured up to 21 days post‐administration. The concentrations of ADC and total antibody in plasma were determined using a validated ligand‐binding assay, with a lower limit of quantification set at 0.02 µg mL^−1^. Briefly, immune plates were coated overnight at 4°C with 1 µg mL^−1^ of human His‐tagged CDH17 protein (Kactusbio, Shanghai, China, CDH‐HM117) in coating buffer. After washing, the plates were blocked, and each serially diluted sample was added to the wells. Following incubation for 1 to 2 h at 4°C, the plates were washed again and incubated with either HRP‐conjugated anti‐human IgG Fc secondary antibody (Abcam, ab99759, RRID:AB_10673762) for total antibody measurement, or a biotin‐labeled anti‐exatecan antibody (Abmart, Shanghai, China, ADC‐Ab0001, RRID:AB_2941023) for ADC measurement. After a reaction period of 1 to 2 h at 37°C, TMB solution (KPL, Gaithersburg, MD, USA, 50‐85‐05) was added directly, or after a 10 min incubation with Streptavidin Protein, HRP (Thermo Fisher Scientific, 21124) at 37°C. The reaction was subsequently stopped with 1 M HCl, and the absorbance at 450 nm in each well was measured using a microplate reader.

### 4.15 ELISA

For the binding assay, immune plates were coated overnight at 4°C with 1 µg mL^−1^ of human, monkey, mouse, and rat His‐tagged CDH17 proteins (Kactusbio) in coating buffer. Following washing, the plates were blocked, and each serially diluted sample was added to the wells. After incubation for 1 to 2 h at 37°C, the plates were washed and subsequently incubated with HRP‐conjugated anti‐human IgG secondary antibody for 1 h at 37°C. After another round of washing, TMB solution was added and incubated at 37°C for 10 min, after which the reaction was halted with 1 M HCl. The absorbance at 450 nm in each well was measured using a microplate reader.

### 4.16 CDH17‐Integrin α2β1 Blocking Assay

Blocking activity of clone 814‐H on the interaction between human CDH17 and integrin α2β1 was determined with a competition assay. Briefly, microtiter plates were coated with recombinant integrin α2β1 to capture CDH17 labeled with biotin. Serial dilutions of clone 814‐H or CDH17 were added to the corresponding wells alongside Biotin‐CDH17. After incubation for 1 to 2 h at 37°C, the plates were washed and subsequently incubated with Peroxidase (HRP)‐SA to bound to the Biotin‐CDH17 for 1 h at 37°C. After another round of washing, TMB solution was added and incubated at 37°C for 10 min, after which the reaction was halted with 1 M HCl. The absorbance at 450 nm in each well was measured using a microplate reader.

### 4.17 Toxicity Studies in Monkeys

AMT‐676 and AMT‐676‐E were administered intravenously to cynomolgus monkeys at intermittent 3‐week intervals. Throughout the study, clinical signs, body weight, food consumption, and clinical pathology were closely monitored. Blood samples were collected at specified time points for hematology, blood chemistry, and pharmacokinetic analysis. Necropsies were conducted 1 week or 3 weeks after the third dose for AMT‐676‐E or AMT‐676, respectively.

### 4.18 Statistics and Data

Statistical analyses were conducted using GraphPad Prism 8.02 (GraphPad Software Inc., San Diego, CA, USA). IC50 and EC_50_ values were determined using a Sigmoid Emax model, with dose dependency assessed by the Spearman rank correlation coefficient hypothesis test. Survival analyses were performed utilizing the Kaplan–Meier method, and differences among groups were evaluated using the log‐rank test. For comparisons between two groups, Student's t‐test was employed, while one‐way ANOVA was used for comparisons involving more than two groups. Two‐way ANOVA (Tukey test) was applied for time‐course analyses of tumor volume. The Student's t‐test was two‐sided, and a *p*‐value of < 0.05 was considered statistically significant. The variances among the groups being compared were assumed to be similar.

## Author Contributions

**W.Y.N**., **R.D.Y**., **H.Y**., and **S.J**.: conducted experimental work, acquired and analyzed data, and wrote the manuscript draft. **L.J.M**. and **Q.Z**. contributed to the in vitro experiments. **L.J.W**. and **C.W.C**. contributed to the expression analysis. **Y.Z**. and **Y.S**. contributed to the ADC conjugation. **J.J.Z**. contributed to the **P.K**. analysis. **S.H**., **Y.X.W**., and **Z.J.L**. contributed to the animal experiments. **C.J.Y**.: contributed to the development of methodology. **H.J.J**. and **W.C.Y**.: contributed to the review and revision of the manuscript. **H.Y.L**., **Y.F.T**., and **X.M**.: supervised and commented on the study. **W.F**. and **L.S.H**.: conceived, designed, and supervised the study; provided funding and resources; revised the manuscript.

## Funding

This work has been supported by the New Cornerstone Science Foundation through the XPLORER PRIZE.

## Ethics Statement

The study design regarding de‐identified human data or tissues was approved by the Institutional Research Ethics Committee. All animal studies were carried out following the protocols approved by the applicable Institutional Animal Care and Use Committee.

## Declaration of Interests

**Jing Shi**, **Ya‐ling Huang**, **Linjie Ma**, **Lijun Wang**, **Caiwei Chen**, **Yixuan Wang**, **Yue Zhang**, **Yi Shen**, **Qing Zhang**, **Jianjian Zhang**, **Yanfang Tang**, **Xun Meng**, and **Shu‐Hui Liu** are Multitude Therapeutics (Shanghai) employees and Yanfang Tang, Xun Meng, and Shu‐Hui Liu hold Multitude stock. The patent applications disclosed included WO2024240227A1 and its patent family.

## Conflicts of Interest

The authors declare no conflicts of interest.

## Supporting information




**Supporting File**: advs77521‐sup‐0001‐SuppMat.pdf.

## Data Availability

The data that support the findings of this study are available from the corresponding author upon reasonable request.
