# Supplementary material for: Discovery and Optimization of AMT‐676, a CDH17‐Targeting ADC for the Treatment of Advanced Gastrointestinal Cancers
Source: Adv Sci (Weinh). 2026 Sep 9:e77521. Online ahead of print. doi: 10.1002/advs.77521 (PMC13555363; doi:10.1002/advs.77521)
Supplement: Supplementary file 1 [file ADVS-9999-e77521-s001.pdf]

Supplementary material

Figure S1. Selection of CDH17 as an ADC target for CRC. Related to Figure 1.
Figure S2. CDH17 expression in human and cynomolgus monkeys. Related to Figure 1.
Figure S3. CDH17 IHC staining in GI cancers. Related to Figure 1.
Figure S4. Toxicity assessment of AMT-676-E. Related to Figure 3.
Figure S5. Body weight, hematology and serum chemistry of AMT-676 and AMT-676-E in cynomolgus monkeys. Related to Figure 3.
Table S1. The PK parameters of AMT-676 in nude mice. Related to Figure 2.
Table S2. The PK parameters of AMT-676-E in nude mice. Related to Figure 2.
Table S3. The PK parameters of AMT-676 in cynomolgus monkeys. Related to Figure 3.
Table S4. The PK parameters of AMT-676-E in cynomolgus monkeys. Related to Figure 3.

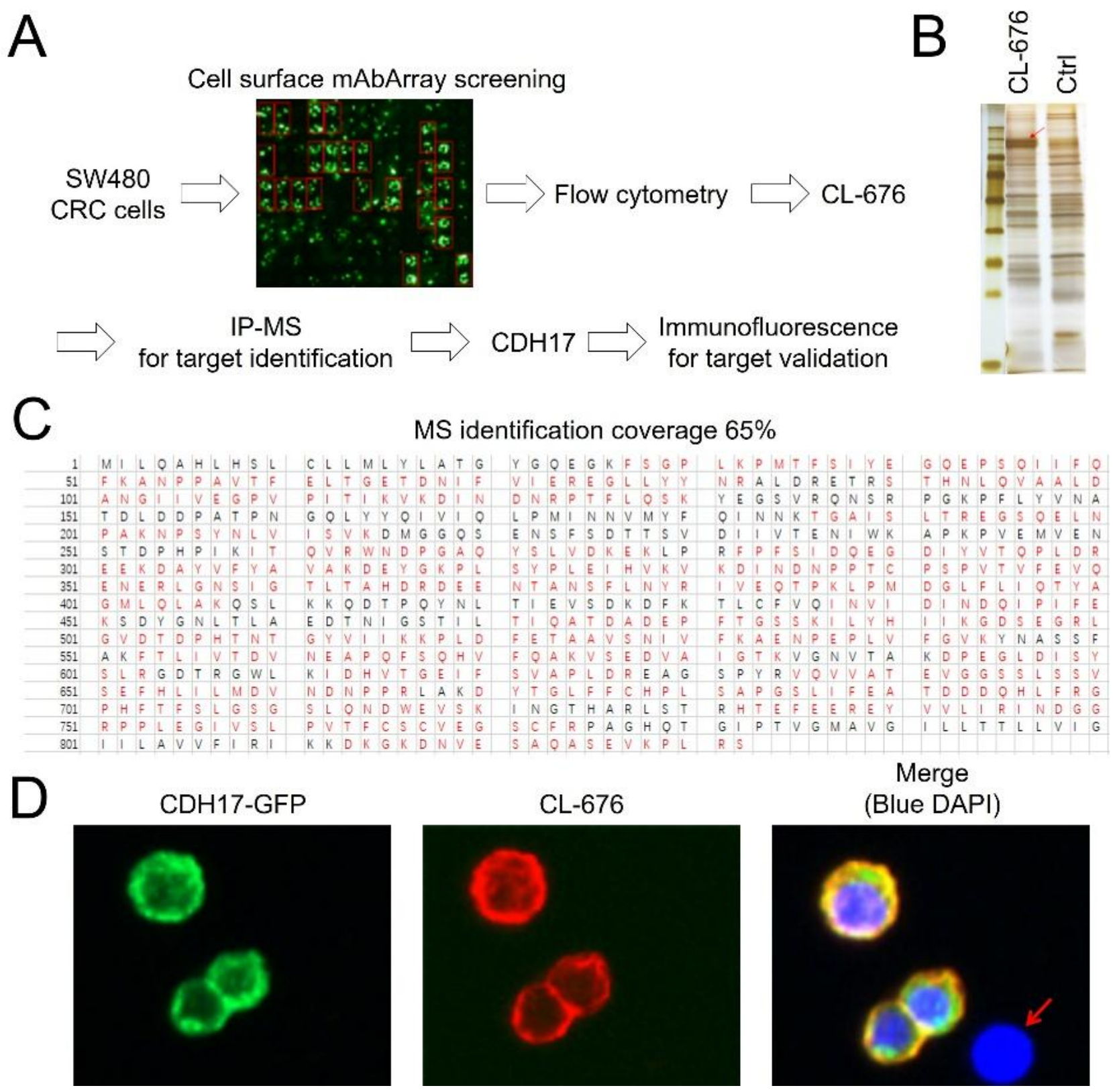

Figure S1. Selection of CDH17 as an ADC target for CRC. Related to Figure1. (A) Overview of the procedure used to identify the target of antibody CL-676. (B) Antigen identification using immunoprecipitation-mass spectrometry (IP-MS) for CL-676. The band indicated by the red arrow was subjected to MS analysis. (C) Peptides identified by mass spectrometry are highlighted in red, with the percentage of all predicted tryptic peptides indicated above. (D) Target validation via immunofluorescence. The binding of CL-676 to CDH17 is demonstrated through immunofluorescence co-localization, illustrating the overlap between red mAb staining and green GFP-tagged CDH17 in 293T cells.

A

Organ	sample number	membrane staining
Cerebrum	3	negative
Cerebellum	3	negative
Adrenal gland	3	negative
Ovary	3	negative
Pancreas	3	negative
Lymph node	3	negative
Hypophysis	3	negative
Testis	3	negative
Thyroid gland	3	negative
Breast	3	negative
Spleen	3	negative
Tonsil	3	negative
Thymus gland	3	negative
Bone marrow	3	negative
Heart	3	negative

Organ	sample number	membrane staining
Esophagus	3	negative
Liver	2	negative
Tongue	3	negative
Kidney	3	negative
Prostate	3	negative
Uterus	3	negative
Cervix	3	negative
Striated muscle	3	negative
Skin	3	negative
Nerve	3	negative
Lung	3	negative
Eye	1	negative
Larynx	3	negative
Stomach	3	-,-,+
Small intestine	3	++, ++, +++
Colon	3	++, ++, +++

B

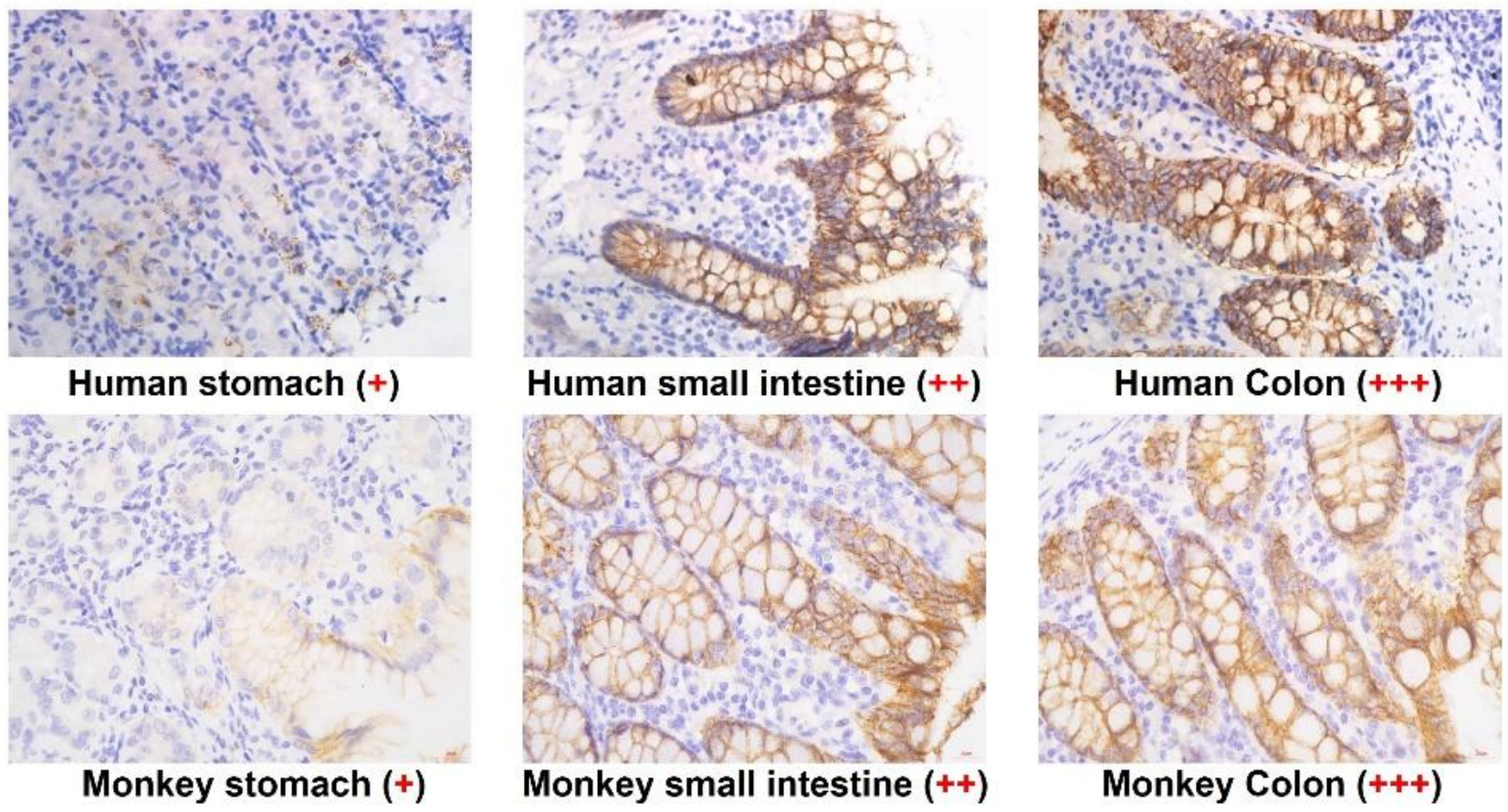

Figure S2. CDH17 expression in human and cynomolgus monkeys. Related to Figure1. (A) Evaluation of CDH17 expression in healthy human organs using IHC staining. (B) Representative images of CDH17 IHC staining in the stomach, small intestine, and colon of both humans and cynomolgus monkeys.

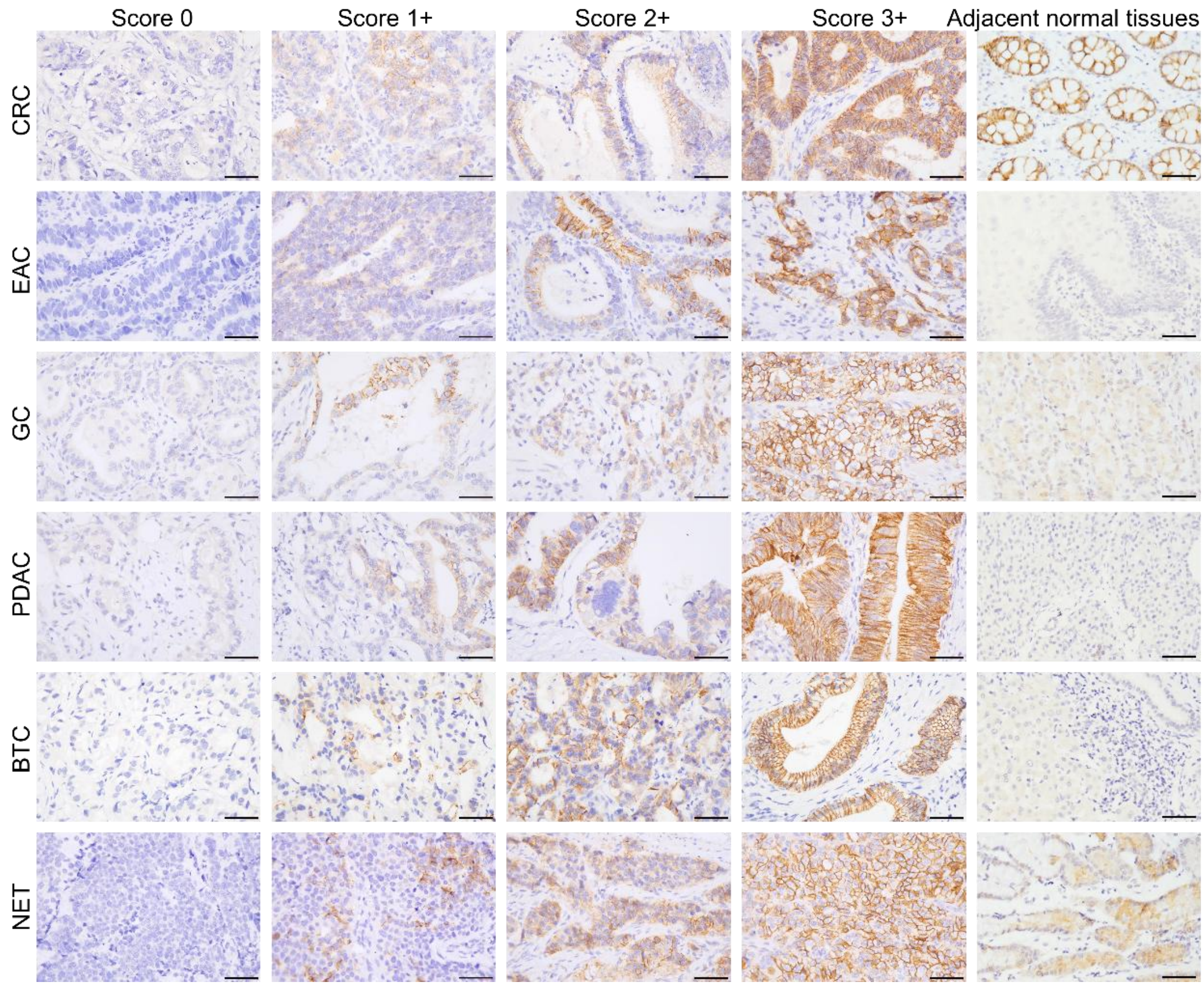

Figure S3. CDH17 IHC staining in GI cancers. Related to Figure 1. Representative images depicting CDH17 staining scores of 0, 1+, 2+, and 3+ in CRC, EAC, GC, PDAC, BTC and NET. Scale bar = 50μM.

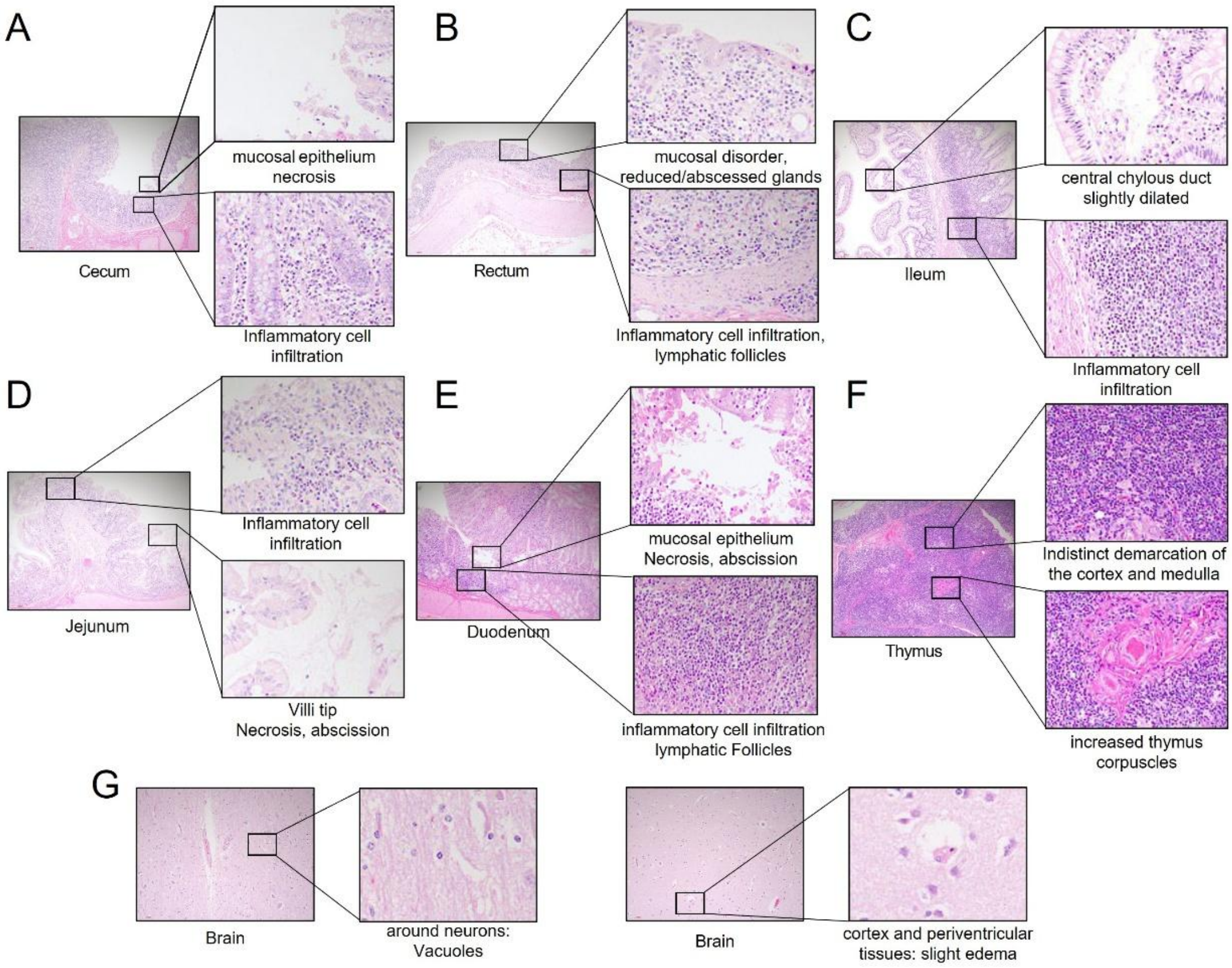

Figure S4. Toxicity assessment of AMT-676-E. Related to Figure 3. Representative H&E staining images illustrating the toxic effects on target organs, including the cecum (A), rectum (B), ileum (C), jejunum (D), duodenum (E), thymus (F), and brain (G), following the administration of AMT-676-E in cynomolgus monkeys.

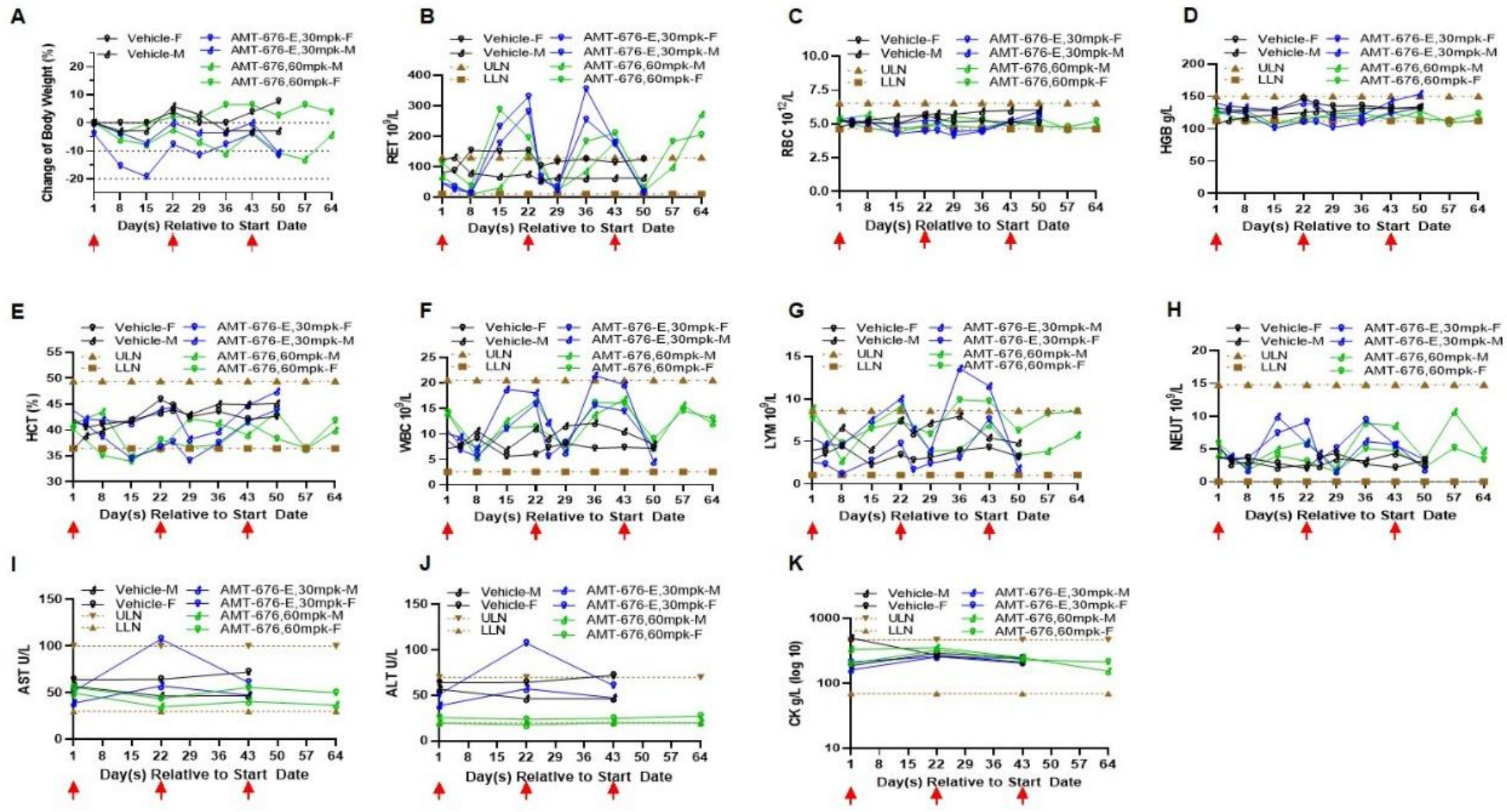

Figure S5. Body weight, hematology and serum chemistry of AMT-676 and AMT-676-E in cynomolgus monkey. Related to Figure 3. Cynomolgus monkeys (one/sex/dose) were administered vehicle, 30, 60, and 80 mg/kg of AMT-676, and 10 and 30 mg/kg of AMT-676-E on days 1, 22, and 43 (indicated by red arrows), followed by a recovery period of 3 weeks for AMT-676 or 1 week for AMT-676-E. Only results for parameters at the HNSTD are presented. (A) Body weight change, with a dashed line indicating the starting body weight. (B-H) Hematology results, including the upper limit of normal (ULN) and lower limit of normal (LLN) for each parameter. (I-K) Serum chemistry results, also showing the ULN and LLN for each parameter.

AMT-676 PK in mice							
	**Parameter**	**Unit**	**Mice #1**	**Mice #2**	**Mice #3**	**Average**	**SD**
**tAb**	**$t_{1/2}$**	**h**	132.8	89.6	126.5	116.3	23.3
	**Tmax**	**h**	0.25	0.25	0.25	0.25	0.00
	**Cmax**	**µg/ml**	98.6	100.1	120.0	106.2	11.9
	**AUC_{0-t}**	**h*µg/mL**	5886.4	4457.1	5683.5	5342.4	773.3
**ADC**	**$t_{1/2}$**	**h**	149.9	74.9	99.4	108.1	38.3
	**Tmax**	**h**	0.25	0.25	0.25	0.25	0.00
	**Cmax**	**µg/ml**	83.0	86.8	114.3	94.7	17.1
	**$AUC_{0-\infty}$**	**h*µg/mL**	4356.5	3441.5	4310.8	4036.2	515.6

Table S1. The PK parameters of AMT-676 in nude mice. Related to Figure 2.

AMT-676-E PK in mice							
	**Parameter**	**Unit**	**Mice #1**	**Mice #2**	**Mice #3**	**Average**	**SD**
**tAb**	**$t_{1/2}$**	**h**	102.9	70.1	122.1	98.3	26.3
	**Tmax**	**h**	0.25	0.25	0.25	0.25	0.00
	**Cmax**	**μg/ml**	106.9	104.2	120.4	110.5	8.7
	**AUC_{0-t}**	**h*μg/mL**	4165.0	3569.2	3761.5	3831.9	304.1
**ADC**	**$t_{1/2}$**	**h**	101.9	91.1	76.1	89.7	13.0
	**Tmax**	**h**	0.25	0.25	0.25	0.25	0.00
	**Cmax**	**μg/ml**	98.6	93.2	76.1	89.3	11.8
	**$AUC_{0-\infty}$**	**h*μg/mL**	3774.2	3554.1	3439.4	3589.2	170.1

Table S2. The PK parameters of AMT-676-E in nude mice. Related to Figure 2.

PK parameters of Non-GLP toxicity study with AMT-676						
	**Dose (mg/kg)**	**Dosing Cycle**	**$t_{1/2}$ (h)**	**Tmax (h)**	**Cmax (μg/mL)**	**$AUC_{0-\infty}$ (h*μg/mL)**
**tAb**	**45**	First	142.50	0.54	1050	112000
		Last	166.50	0.08	1120	132000
	**60**	First	101.00	0.08	1560	124000
		Last	100	0.08	1520	153000
**ADC**	**45**	First	106.05	3.50	1190	85400
		Last	117.00	0.08	1350	91000
	**60**	First	81.6	0.08	1880	96400
		Last	78.6	0.08	1620	114000
	**Dose (mg/kg)**	**Dosing Cycle**	**$t_{1/2}$ (h)**	**Tmax (h)**	**Cmax (ng/mL)**	**$AUC_{0-\infty}$ (h*ng/mL)**
Exatecan	45	First	109	25.5	1.97	321
		Last	102	24	2.00	332
	60	First	82.8	3.5	4.14	538
		Last	78.7	24	3.45	532

Table S3. The PK parameters of AMT-676 in cynomolgus monkeys. Related to Figure 3.

PK parameters of Non-GLP toxicity study with AMT-676-E						
	**Dose (mg/kg)**	**Dosing Cycle**	**$t_{1/2}$ (h)**	**Tmax (h)**	**Cmax (μg/mL)**	**$AUC_{0-\infty}$ (h*μg/mL)**
**tAb**	**10**	First	36.38	0.08	223.40	11108.65
		Last	50.33	0.08	146.28	7359.92
	**30**	First	37.44	0.08	588.45	28908.44
		Last	34.39	2.00	442.50	22169.30
**ADC**	**10**	First	36.60	0.08	286.44	13480.92
		Last	22.00	0.08	176.59	6497.25
	**30**	First	40.35	2.00	569.10	31950.28
		Last	32.92	2.00	463.88	21724.41
	**Dose (mg/kg)**	**Dosing Cycle**	**$t_{1/2}$ (h)**	**Tmax (h)**	**Cmax (ng/mL)**	**$AUC_{0-\infty}$ (h*ng/mL)**
**Exatecan**	**10**	First	52.41	24	2.62	240.83
		Last	61.83	48	1.50	102.73
	**30**	First	69.79	15	9.06	651.13
		Last	55.06	4	4.51	452.41

Table S4. The PK parameters of AMT-676-E in cynomolgus monkeys. Related to Figure 3.
